# Spatial Factors Shape Taxonomic and Functional Beta‐Diversity in Water‐Filled Tree Holes in Different Biogeographical Regions

**DOI:** 10.1111/ele.70294

**Published:** 2025-12-28

**Authors:** Francesca Cerroti, Thibaut Rota, Francisco Valente‐Neto, K. T. Fahis, Red Calore, Gustavo Q. Romero, Karumampoyil Sakthidas Anoop Das, Andreas Bruder, Martin M. Gossner

**Affiliations:** ^1^ Institute of Microbiology Scuola Universitaria Professionale della Svizzera Italiana (SUPSI) Mendrisio Switzerland; ^2^ Forest Entomology Swiss Federal Institute for Forest, Snow and Landscape Research WSL Birmensdorf Switzerland; ^3^ Department of Environmental Systems Science Institute of Terrestrial Ecosystems, ETH Zürich Zürich Switzerland; ^4^ Instituto de Biologia, Departamento de Biologia Animal Universidade Estadual de Campinas (UNICAMP) São Paulo Brazil; ^5^ Center for Conservation Ecology & Department of Zoology M.E.S Mampad College (Autonomous – Calicut University) Malappuram Kerala India

**Keywords:** beta‐diversity, community assembly, environmental factors, functional diversity, metacommunity dynamics, richness differences, spatial factors, taxonomic diversity, turnover, water‐filled tree holes

## Abstract

A central goal in ecology is to understand the mechanisms shaping community assembly at different spatial and temporal scales. This knowledge is crucial for improving conservation strategies, but remains limited for ephemeral habitats. We investigated the contribution of environmental, that is, physical and chemical microhabitat properties, and spatial factors in shaping taxonomic and functional β‐diversity of macroinvertebrate metacommunities inhabiting water‐filled tree holes (WTHs) in forests in three biogeographical regions: Temperate‐Mediterranean (France), Neotropical (Brazil), and Palaeotropical (India). We conducted standardised surveys of 35 WTHs per region on 100 ha plots. Spatial factors had a stronger effect on taxonomic and functional β‐diversity than environmental properties. Species richness differences dominated taxonomic β‐diversity. Processes driving functional β‐diversity showed biogeographic patterns, with functional turnover being pronounced in the Palaeotropical rainforest. These findings highlight the key role of spatial processes in shaping WTH metacommunities and emphasise the need for conservation strategies that maintain habitat connectivity and old‐growth forests.

## Graphical abstract

Spatial processes predominantly shape taxonomic and functional β‐diversity of macroinvertebrate communities in water‐filled tree holes in different biogeographical regions, with species richness differences driving both taxonomic and functional patterns overall, while functional turnover was pronounced in the Palaeotropical region. These findings highlight the importance of maintaining habitat connectivity and old‐growth forests for biodiversity conservation.

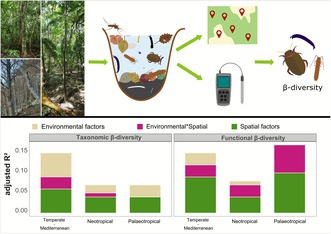

## 1 Introduction

Understanding how biodiversity is distributed and what processes govern community assembly is a core question in ecology (Mittelbach and McGill 2019). Niche‐based theories emphasise deterministic mechanisms such as environmental filtering and biotic interactions (Wilson and Gitay 1995), whereas neutral theory highlights the importance of stochasticity, including ecological drift, stochastic colonisation and extinction dynamics under dispersal limitation, and priority effects (Hubbell 2011). These perspectives are integrated in the metacommunity framework, which conceptualises biodiversity patterns as the result of deterministic and stochastic processes across scales (Leibold et al. 2004). Over the past two decades, this framework has provided a powerful approach for examining how environmental gradients, dispersal constraints, and colonisation‐extinction dynamics shape community composition (Heino 2011; Mouquet and Loreau 2003). However, despite its broad application, there is no clear consensus on the relative importance of environmental and spatial drivers of community variation across environmental contexts and biogeographic regions (Leibold and Chase 2017; Soininen et al. 2018; Xu et al. 2023).

A key approach to understanding how environmental and spatial processes shape metacommunity dynamics is the analysis of β‐diversity: the variation in species composition among sites or habitat patches within a region (Leibold et al. 2004; Whittaker 1972). By linking local and regional diversity, β‐diversity captures spatial patterns in community structure and provides insights into underlying assembly mechanisms (Leibold et al. 2004; Mori et al. 2018). Traditionally, β‐diversity has been assessed taxonomically, focusing on differences in species identities across sites (Ricklefs 1987). However, taxonomic composition overlooks ecological equivalence, as functionally similar species may replace one another without altering ecosystem processes. To address this, recent works have expanded β‐diversity analyses to include functional diversity based on traits that influence species' ecological roles, such as morphology, physiology and behaviour (Fu et al. 2019; Hu et al. 2023; Si et al. 2016). Because functional trait composition often responds more directly to environmental gradients, it can offer deeper mechanistic insights than taxonomic composition alone (Guzman et al. 2021; Soininen et al. 2016; Srivastava et al. 2023). Examining both taxonomic and functional diversity thus provides a more complete understanding of community assembly (Heino and Tolonen 2017; Meynard et al. 2011).

As research on β‐diversity advances, partitioning it into its two components, species turnover and richness differences, has become essential for understanding community assembly processes and, at the same time, provides valuable insights for guiding conservation strategies (Baselga 2010; Legendre 2014). Turnover reflects species replacement between sites, often driven by environmental filtering and biotic interactions; when this component predominates, conservation should focus on a regional approach that protects a network of multiple sites. In contrast, richness differences arise from variations in species number due to dispersal limitation or environmental fluctuations; in such cases, prioritising sites with high local diversity is likely to be more effective (Baselga 2010; Harrison et al. 1992; Hill et al. 2019). Furthermore, these components likely represent distinct assembly processes whose relative importance varies with environmental stability (Gianuca et al. 2017; Hill et al. 2017; Si et al. 2016).

In ecosystems with relatively stable climatic conditions throughout the year, such as tropical rainforests, turnover often dominates β‐diversity, as climatic stability promotes species specialisation and together with high environmental heterogeneity, fosters niche differentiation (Leibold et al. 2004; Soininen et al. 2018). At the same time, biotic interactions such as competition and predation are considered stronger in low‐latitude ecosystems (Schemske et al. 2009; Willig et al. 2003), further reinforcing species replacement across sites. Together, environmental specialisation and intense biotic interactions are expected to enhance turnover in the tropics (Drakare et al. 2006; Hillebrand 2004; Soininen et al. 2007, 2018). In contrast, in regions with stronger temporal climatic variation, including Mediterranean and temperate forests, richness differences are expected to play a larger role. Fluctuating abiotic conditions such as droughts or frosts periodically alter resource availability and species performance. These fluctuations weaken deterministic niche processes by interrupting consistent environmental filtering and preventing the long‐term establishment of competitive dominance. Consequently, community dynamics are increasingly driven by stochastic processes such as dispersal limitation and colonisation‐extinction events (Baselga 2010; Leprieur et al. 2011; Soininen et al. 2018).

Most research on β‐diversity and its components in freshwater ecosystems has focused on perennial habitats, including lakes, permanent ponds and streams (Aspin et al. 2018; Datry et al. 2016). As a consequence, dynamic environments such as temporary ponds, intermittent streams, rock pools and phytotelmata remain underrepresented in metacommunity research (Datry et al. 2016; Logue et al. 2011; Vanschoenwinkel et al. 2013; Wisnoski et al. 2019), despite their broad distribution and ecological significance (Acuña et al. 2014; Shipley et al. 2023; Srivastava et al. 2023). In these systems, short hydroperiods, drying events, and climatic variability are expected to shift the balance of community assembly toward stochasticity, potentially increasing the contribution of richness differences to β‐diversity. Yet, this prediction has rarely been tested with standardised, replicated field studies across environmental contexts (Gianuca et al. 2017; Hill et al. 2017; but see Penna et al. 2025). Addressing this gap is critical for identifying general rules of taxonomic and functional community assembly and informing biodiversity conservation in increasingly variable environments (Heino, Melo, Siqueira, et al. 2015).

Water‐filled tree holes (WTHs) provide an ideal model system to study how environmental and spatial processes shape taxonomic and functional β‐diversity and its components (Srivastava et al. 2004). These ephemeral, spatially discrete aquatic habitats form in tree cavities across tropical and temperate forests and are inherently unstable due to their limited volume and susceptibility to frequent drying (Petermann and Gossner 2022). A key strength of WTHs as a model system is their substantial environmental variation. Even within a single region or location, WTHs can differ greatly in abiotic conditions such as conductivity, nutrient availability, and organic matter content (Gossner et al. 2016). This environmental heterogeneity is further shaped by disturbance regimes differing across regions. Hydroperiods are typically shorter and more variable in Temperate‐Mediterranean forests due to recurrent drying and freezing, whereas in Paleotropical and Neotropical forests they are longer and more stable, interrupted mainly by seasonal desiccation but reliably replenished by heavy rains or monsoons (Gossner 2018; Kitching 2000; Nishadh and Das 2014, Figure S1). These differences provide a unique setting for examining how environmental filtering influences β‐diversity distributions. At the same time, WTHs are embedded in landscapes where dispersal among habitat patches is constrained by geographic distance (Petermann and Gossner 2022), providing opportunities to test the role of spatial processes. Organisms inhabiting WTHs disperse through multiple strategies: active flight or crawling, passive transport by wind or rain, and phoresy on vertebrates such as frogs, birds, or small mammals (Kitching 1971), which shape the interconnectedness of communities between WTHs. Together, the discrete nature, strong environmental variation, and spatial structuring of WTHs make them well suited for disentangling the relative and combined effects of environmental and spatial drivers on community assembly processes in ephemeral ecosystems.

With this study, we aimed to improve our understanding of (1) the relative importance of the two components of β‐diversity, richness differences and turnover, in shaping both taxonomic and functional community composition in WTH, and (2) how environmental and spatial factors influence these components in WTH communities distributed across biogeographical regions that differ in climate, structural complexity, and environmental stability. This comparative approach allowed us to assess the extent to which community assembly processes follow general patterns, or are contingent on characteristics of particular biogeographical regions (Lawton 1999). We first hypothesized that species richness differences will generally play a dominant role in shaping β‐diversity across biogeographic regions (H1). Richness differences tend to prevail in unstable environments, where frequent environmental fluctuations weaken deterministic processes, thereby increasing the relative influence of stochastic dynamics like dispersal limitation and colonisation‐extinction events. Additionally, we hypothesized that spatial factors, representing broad to fine‐scale patterns within our locations, will be the primary drivers of community variability in WTHs across biogeographic regions (H2). This expectation reflects the generally small size and ephemeral nature of these systems, where frequent disturbances such as freezing, drying and refilling are expected to enhance the importance of dispersal and colonisation‐extinction dynamics. As environmental instability is expected to be particularly pronounced in the Temperate‐Mediterranean biogeographic region, where high seasonality occurs, we predicted that both the influence of spatial processes (H2) and the dominance of richness differences (H1) would be most pronounced in this region. We formulated the same hypothesis for both taxonomic and functional β‐diversity, based on the expectation that ecological processes such as environmental filtering and colonisation‐extinction dynamics are reflected by both species identities and their ecological traits (Swenson 2014). To test these hypotheses, we conducted a replicated field study, sampling 105 WTHs in three forests (i.e., 35 in each) across three biogeographic regions, that is, Temperate‐Mediterranean, Neotropical, and Palaeotropical. We focused on primeval forests, or forests that have not been managed for more than a hundred years, to ensure that natural dynamics prevailed.

Our findings highlight the key role of spatial processes in shaping both taxonomic and functional β‐diversity in WTH communities, underscoring the importance of maintaining WTH connectivity and integrity for forest conservation.

## 2 Material and Methods

We carried out the study in three old‐growth forests located in distinct biogeographical regions. The Massane beech forest in the Oriental Pyrenees, France (42°28′58″ N, 3°1′45″ E), is part of a UNESCO World Natural Heritage site. The Amazonian rainforest site is a 7000 ha conservation area in Cotriguaçu, Mato Grosso, Brazil (9°48′ S, 58°16′ W). The Kakkadampoyil rainforest is situated in the Western Ghats, a biodiversity hotspot in Kerala, India (11°19′03″ N, 76°08′15″ E; Myers et al. 2000). These sites represent the Temperate‐Mediterranean, Neotropical, and Palaeotropical biogeographical regions and differ in elevation, temperature, and precipitation (Table S1). In each biogeographical region, we selected a relatively pristine and accessible forest. Within each forest, we established a 100 ha plot located at least 100 m from the forest edge (hereafter “site”). Mapping and sampling of WTHs were conducted between 4 and 12 April 2022 in the Temperate‐Mediterranean forest, 9–21 January 2023 in the Neotropical forest, and 24 October to 2 November 2023 in the Palaeotropical forest. These periods were chosen to coincide with hydrologically favourable conditions in each forest (spring rainfall in the Temperate‐Mediterranean, the rainy season in the Neotropical, and the post‐monsoon phase in the Palaeotropical, Figure S1), when WTHs were full of water and communities were reactivated from dormant stages, thus ensuring ecological comparability despite different climatic regimes and sampling times.

At each site, we recorded the geographical position of each WTH, using a GPS (Garmin GPSMAP65). We conducted the mapping after the wet season, so all the WTH were filled with rainwater. Of the mapped WTHs, we randomly selected 35 to be sampled using a stratified random design. This stratified sampling aimed at including a balanced number of various types of WTHs: (i) pan and rot holes, (ii) ground holes, below two meters, and canopy holes, above two meters (Kitching 1971). Dry holes and small holes (< 200 mL) were excluded from sampling. However, due to the special characteristics of the forests studied, a completely balanced design was not possible at all sites. Ground‐level, rot, and pan holes dominated in the Palaeotropical, Neotropical and Temperate‐Mediterranean sites, respectively.

We assessed macroinvertebrate community composition by sampling all 35 WTHs per site following Yanoviak and Fincke (2005). We extracted water using a syringe connected to a pipe and manually collected remaining organic matter with spoons adapted to WTH morphology. At each field station, we processed samples in three steps: (i) flushed material through sieves of 0.5 cm, 500 μm and 300 μm; (ii) classified detritus ≥ 0.5 mm as coarse particulate organic matter (CPOM) and the remainder as fine particulate organic matter (FPOM); and (iii) searched all fractions for macro‐organisms, preserving macroinvertebrates in 70% ethanol while identifying, counting, and releasing Chordata. We estimated organism abundance in FPOM from one‐third of the sediment volume and extrapolated it to total volume. We identified aquatic macroinvertebrates and Chordata to the lowest possible taxonomic level, accounting for limited knowledge of tropical fauna and assigned them to morphospecies (Cranston et al. 1987; Cranston 1982; Klausnitzer 1996, 2025; Thorp et al. 2018). We barcoded one or two individuals of each arthropod morphospecies (COI) to verify identification. Finally, we oven‐dried both organic matter fractions for ~48 h at 40°C and weighed them for use as environmental predictors.

The traits of invertebrates colonising WTHs are not well known, especially in the tropics (Petermann and Gossner 2022). However, freshwater macroinvertebrates inhabiting WTHs share several ecological traits, at higher taxonomic levels, with those occurring in other phytotelmata such as tank bromeliads. Similar to WTHs, tank bromeliads are water‐filled microcosms reliant on detrital inputs and subject to similar constraints, including limited space and fluctuations in water and oxygen availability (Kitching 2000; Srivastava et al. 2004). We used an existing database of macroinvertebrate functional traits, compiled for tank bromeliad invertebrates (Céréghino et al. 2018) to assign traits to the macroinvertebrates in our study. We included seven trait groups: aquatic developmental stage, maximum body size, locomotion, dispersal mode, respiration mode, feeding group, and cohort production interval. Each nominal trait comprised multiple modalities or states, as detailed in Table 1 of Céréghino et al. (2018).

**TABLE 1:** β‐diversity and contributions of its components for each location.

Taxonomic					
Biogeographical region	*β*	D	T	D/β	T/β
Temperate‐Mediterranean	0.43	0.3	0.13	0.7	0.3
Neotropical	0.41	0.26	0.15	0.65	0.34
Palaeotropical	0.31	0.22	0.09	0.67	0.3
Functional					
Temperate‐Mediterranean	0.3	0.21	0.09	0.7	0.3
Neotropical	0.19	0.12	0.07	0.63	0.37
Palaeotropical	0.13	0.06	0.07	0.42	0.58

Abbreviations: β, denotes the total β‐diversity; D, species richness difference; D/β, is the proportion of total β which is explained by species richness difference; T, is species turnover; T/β, is the proportion of total β which is explained by species turnover.

For each WTH we recorded structural variables: current and potential water depth, height above ground, opening size, hole origin (stem fork, branch break, stem break, stem hole, root hole), orientation (16‐point compass direction), circumference at breast height, WTH type (pan or rot hole), and water volume at sampling. We measured physico‐chemical parameters with a HACH HQ Series probe, including oxygen concentration and saturation, water temperature, pH and conductivity. We collected 50 mL mixed water samples and analysed ammonium (flow injection with gas diffusion/photometric detection), nitrogen, total phosphate, sulfate and chloride (ion chromatography). We measured CDOM (μg/L), chlorophyll‐a (μg/L, AquaFluor) and turbidity (FNU, HACH 2100Q) after 0.45 μm filtration. Because we could not measure these three variables in the Temperate‐Mediterranean forest, we repeated analyses without them; as results remained consistent, we retained them in the final dataset. We also quantified dry organic matter (FPOM, CPOM, and total).

All analyses were conducted separately for each forest site, using R version 4.2.1 (R Core Team 2022).

We used Moran's eigenvector maps (MEM; Dray et al. 2006) with the adespatial package (Dray et al. 2023; Griffith and Peres‐Neto 2006) to generate orthogonal and linearly independent spatial variables for each biogeographic region. We first defined two matrices: (1) a binary connectivity matrix A based on a Gabriel graph, where two sites connect if no other site lies within the circle defined by their distance, and (2) a distance‐based weighting matrix B:

$$w_{\mathrm{ij}}=1-\frac{d_{\mathrm{ij}}}{\max\left(d\right)}$$

where *d*_*ij*_ is the Euclidean distance between WTHs *i* and *j*, and max (d) is the maximum distance between any two WTHs. These procedures assign higher weights to closer WTHs. We then combined A and B by element‐wise (Hadamard) product to obtain the spatial weighting matrix W. We subjected W to Principal Coordinate Analysis (PCA), using the resulting eigenvectors (MEMs) as spatial explanatory variables (Borcard and Legendre 2002). MEMs with lower eigenvalues represent broad‐scale spatial structures, while higher eigenvalues represent fine‐scale patterns (Figure S2; Griffith and Peres‐Neto 2006).

In parallel, we assessed the connectivity of each sampled and mapped WTH through network analysis. The Gabriel graph was converted into an igraph object using the graph_from_adj_list function, and node degree (i.e., number of connections) was calculated with the degree function in the igraph package (Csardi and Nepusz 2006). Degree values for each WTH were compiled into a separate data frame for further analysis.

To avoid multicollinearity among environmental predictors, we first analysed the correlations among predictors by calculating Spearman correlation coefficients. Following Tabachnick and Fidell (2019), we excluded variables with bivariate correlations > 0.70. Among highly correlated variables, we retained those most accurate and relevant for describing WTH characteristics. We *z*‐standardised all environmental predictors (centered to mean = 0 and scaled to unit variance) using the ‘standardise’ method in the decostand function of the vegan package (Oksanen et al. 2022).

To analyse taxonomic β‐diversity, we examined WTHs‐by‐species matrices containing abundance data. Pairwise dissimilarities were calculated using percentage difference as dissimilarity index (also known as Bray‐Curtis index). This analysis was further refined by decomposing β‐diversity into its components: species richness differences and species turnover. We used the Podani family decomposition method for this purpose, as it generates Euclidean matrices that can be directly represented in Euclidean space (Legendre 2014). The decomposition was performed with the beta.div.comp function from the adespatial package (Dray et al. 2023).

We assessed functional β‐diversity using hyperdimensional functional trait spaces that capture the diversity of ecological roles within assemblages. We derived these trait spaces with kernel density estimation of hypervolumes following Mammola and Cardoso (2020). For each WTH community, we applied equal weighting to all functional traits and used the Gower distance metric to quantify dissimilarities between species based on multivariate functional traits, accommodating both continuous and categorical data. We estimated the 95% density of stochastic points within each trait space using the Gaussian method implemented in the kernel. build function of the BAT package (Cardoso et al. 2024), generating probabilistic hypervolumes that characterise functional β‐diversity. We incorporated abundance data and reduced dimensionality to three axes to facilitate interpretation. Finally, we partitioned β‐diversity into turnover and net difference in amplitude components of n‐dimensional hypervolumes, representing functional richness differences, using the kernel. beta function in the BAT package (Cardoso et al. 2024).

To select the significant explanatory variables associated with β‐diversity, we performed a forward selection procedure for both taxonomic and functional β‐diversity, based on the highest adjusted *R* ^2^ value. Then, 999 permutations were computed to determine the minimal set of variables that significantly and independently influenced β‐diversity. This involved using each dissimilarity matrix individually as response data and the environmental and spatial variables separately as explanatory predictors (Legendre 2014).

We used the distance‐based redundancy analysis (db‐RDA) method (Legendre and Anderson 1999) to explore the relationships between variation in assemblage dissimilarity matrices (total β‐diversity, richness differences, turnover), environmental and spatial predictors at the three forest sites for both taxonomic and functional diversity. We performed the db‐RDA using the dbRDA.D function from appendix S4 in Legendre (2014), which provides an *F*‐test to determine the significance of the response data matrix (β‐diversity and its components) in relation to a set of explanatory variables (environmental and spatial).

We applied variance partitioning to estimate the relative contribution of selected environmental and spatial predictors on each of the dissimilarity matrices (Peres‐Neto et al. 2006). This approach, widely used in ecology, evaluates the significance of environmental and spatial variables in metacommunities (Clappe et al. 2018). The analyses were conducted separately for both taxonomic and functional β‐diversity and their components. To assess the significance of each component, we conducted an ANOVA‐like test. These analyses were implemented using the varpart and anova functions from the vegan package (Oksanen et al. 2022). To summarise their relative importance across biogeographical regions and β‐diversity components, we report the number of times each factor explained a greater variation, a descriptive comparison based on model outputs.

## 3 Results

We recorded 2214 individuals (10 morphospecies) in the Temperate‐Mediterranean forest, 5701 (20 morphospecies) in the Neotropical forest, and 31,004 (21 morphospecies) in the Palaeotropical forest. Local richness per WTH ranged from 1 to 9 (mean 4 ± 1.8 SD), 2–11 (6 ± 2.65 SD) and 5–15 morphospecies (10 ± 2.55 SD), respectively (Table S2). We recorded different densities of WTHs per hectare: 75 in the Temperate‐Mediterranean forest, 135 in the Neotropical forest and 172 in the Paleotropical forest.

Environmental variables varied widely among WTHs. In the Temperate‐Mediterranean forest, nitrate and phosphate had very high coefficients of variation (233%, 432%) (Table S3). In the Neotropical forest, conductivity and sulfate were most variable (308%, 229%). In the Palaeotropical forest, chlorophyll‐a and water volume varied the most (210%, 201%). After removing collinearity (Table S4a–c), 15–18 forest‐specific predictors remained for forward selection of taxonomic and functional β‐diversity. Spatial eigenfunctions yielded 35 MEMs for the Temperate‐Mediterranean forest, 32 for the Neotropical forest and 34 for the Palaeotropical forest, capturing broad (MEM1‐11/12), medium (MEM12/13‐23/25), and fine (MEM24/26‐32/35) spatial scales. Different MEMs were selected per β‐diversity component (Tables S5 and S6).

**FIGURE 1:**
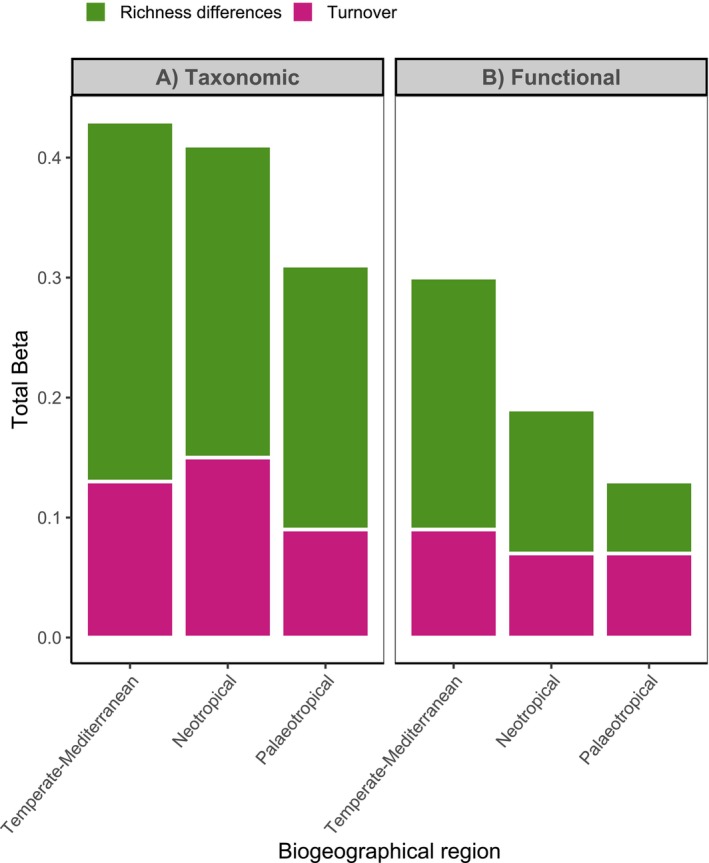
Taxonomic (A) and functional (B) β‐diversity, showing the contributions of its two components: richness differences (in green) and turnover (in pink) for each studied biogeographical region.

Taxonomic β‐diversity ranged from 0.31 to 0.43 across all sites, with species richness differences accounting for approximately 70% of the total β‐diversity (Figure 1, Table 1). Functional β‐diversity ranged from 0.13 to 0.3 (Figure 1, Table 1). While functional richness difference dominated functional β‐diversity in the Temperate‐Mediterranean and Neotropical forests, functional turnover was most important in the Palaeotropical forest. The dominant components accounted for approximately 60% of the total β‐diversity (Figure 1, Table 1).

**FIGURE 2:**
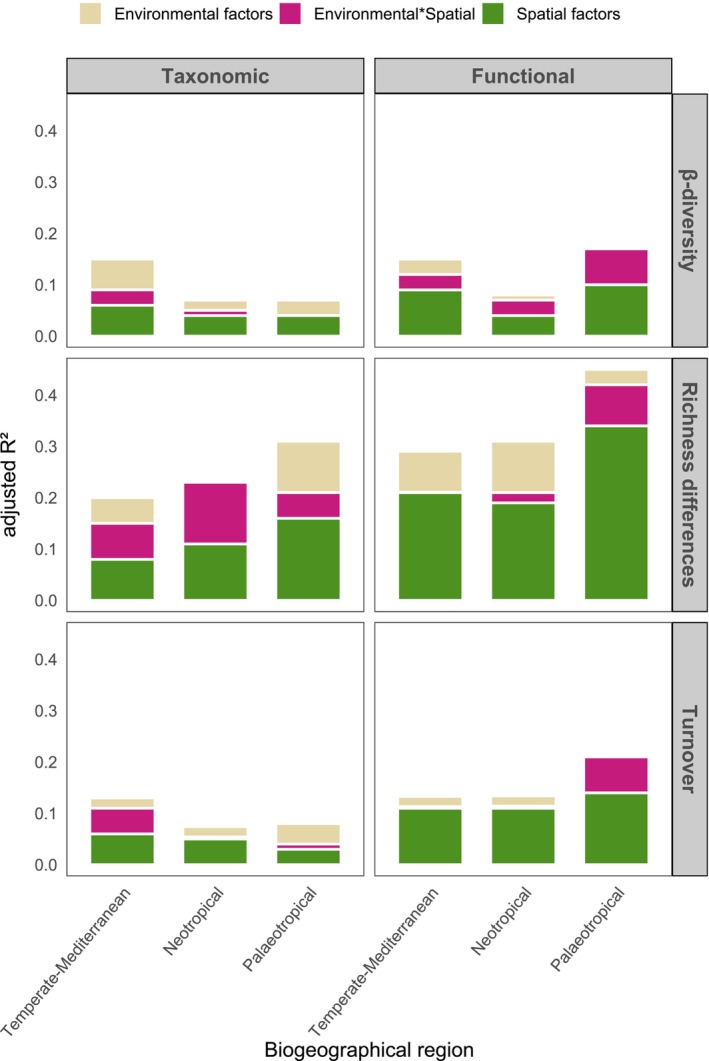
Variation partitioning results showing the unique and shared effect of spatial and environmental factors on taxonomic (left side) and functional (right side) β‐diversity and its components for the different studied biogeographical regions.

According to the results of the dbRDA, different sets of environmental and spatial factors were identified as key predictors of individual β‐diversity dimensions and biogeographic regions (Tables S5 and S6, Figures S3–S8). Variation partitioning revealed that spatial factors were generally more important than environmental variables in structuring the two dimensions of β‐diversity and its components. For taxonomic β‐diversity, pure spatial factors significantly (*p* < 0.05) explained a greater portion of variation than pure environmental factors in 7 out of 9 biogeographical regions × β‐diversity component combinations, indicating a generally consistent spatial structuring of community composition across sites and components. Pure environmental factors explained less variation overall and were not significant in three cases: richness differences in the Neotropical and Temperate‐Mediterranean forests and species turnover in the Temperate‐Mediterranean forest (Figure 2, Table S7). For functional β‐diversity, pure spatial factors significantly (*p* < 0.05) explained the largest portion of variation in all 9 biogeographical regions × component combinations, highlighting a consistently dominant role of spatial processes across biogeographical regions and β‐diversity components. In contrast, pure environmental factors contributed less to the explained variation and were only significant in three cases: richness differences in the Neotropical biogeographical and Temperate‐Mediterranean forests and turnover in the Neotropical forest (Figure 2, Table S8).

## 4 Discussion

Our study provides novel insights into the importance of environmental and spatial processes for community assembly in ephemeral habitats, here water‐filled tree holes, across distinct biogeographical regions. Although each region was represented by a single forest type, limiting broad‐scale inference, the selected forests are characteristic of their respective biogeographical regions and likely capture the dominant patterns of the surrounding forest landscape. Taxonomic β‐diversity was predominantly shaped by differences in species richness rather than species turnover, which is consistent with colonisation‐extinction dynamics in these ephemeral habitats, supporting our first hypothesis (H1). This pattern was especially pronounced for taxonomic β‐diversity, where richness differences accounted for approximately 70% of the total dissimilarity. Functional β‐diversity exhibited biogeographic‐specific variation. Functional richness differences dominated in the Neotropical and Temperate‐Mediterranean forests, whereas functional turnover played a greater role in the Palaeotropical forest. In line with our second hypothesis (H2), spatial factors were the dominant drivers of functional β‐diversity and its components across all biogeographical regions, and also explained more variation than environmental factors in most cases for taxonomic β‐diversity. These findings highlight the strong influence of spatial processes on WTH community composition, while also suggesting that biogeographical region‐specific environmental conditions may contribute to shaping functional community structure.

The observed dominance of taxonomic richness differences, contributing approximately 70% to overall taxonomic β‐diversity in each biogeographical region, emphasises the generality of community assembly processes in these habitats. This result aligns with previous studies showing that environmental instability in ephemeral habitats promotes local extinctions and recolonisations, leading to compositional variations among habitat patches driven by species richness differences rather than species turnover (Baselga 2010; Dobrovolski et al. 2012; Jansson 2003; Soininen et al. 2018). Taxonomic turnover remained limited, although our studied WTHs exhibited high environmental variability suggesting that species‐specific differences in dispersal capabilities, stochastic dispersal dynamics and habitat instability primarily shaped β‐diversity (Heino, Melo, Siqueira, et al. 2015; Soininen et al. 2013).

The consistent dominance of taxonomic richness differences across biogeographic regions was unexpected. A recent meta‐analysis showed that species turnover is generally higher in tropical freshwater ecosystems due to greater environmental stability, habitat heterogeneity and more complex biotic interactions (Soininen et al. 2018). The absence of this pattern in our study likely reflects the fact that WTHs are ephemeral habitats, where frequent disturbances and short hydroperiods disrupt deterministic community assembly processes. Regardless of the biogeographic region, WTHs seem to be governed by disturbance events that promote stochastic recolonization rather than deterministic species sorting. As seen in other temporary aquatic systems, such as rock pools and seasonal ponds, taxonomic richness differences tend to dominate when community composition is often reset by extreme events such as droughts or floods (Florencio et al. 2014; Heino, Melo, Siqueira, et al. 2015; Soininen et al. 2013).

Functional β‐diversity exhibited biogeographic‐specific patterns. While richness differences dominated in the Neotropical and Temperate‐Mediterranean forests, functional β‐diversity in the Palaeotropical forest was primarily driven by functional turnover. This was unexpected given that both the Neotropical and Palaeotropical forests are tropical rainforests with relatively stable climate conditions, compared to the Temperate‐Mediterranean forest, under which we expected high functional turnover due to higher environmental heterogeneity and niche differentiation (Drakare et al. 2006; Hillebrand 2004; Soininen et al. 2007). The fact that only the Palaeotropical forest followed this expectation suggests that additional factors, such as fine‐scale environmental heterogeneity, intense biotic interactions, or distinct evolutionary histories, may amplify functional divergence in that region (Mouquet and Loreau 2003; Soininen et al. 2018). The pronounced functional turnover may reflect the unique evolutionary context in the southern Western Ghats, shaped by long‐term climatic stability (Page and Shanker 2020) and prolonged continental isolation following India's drift from Gondwana (Joshi and Karanth 2013). In contrast, the dominance of richness differences in the Neotropical and Temperate‐Mediterranean forests indicates a greater role for stochastic colonisation‐extinction dynamics, where functionally similar species are replaced across WTHs (Baselga 2012). These results highlight that even structurally and climatically similar ecosystems, here Neotropical and Palaeotropical rainforests, can diverge in the processes shaping functional β‐diversity, emphasising the influence of historical and regional contingencies on community assembly.

Spatial factors explained more variation than environmental variables for functional β‐diversity in all cases, and for taxonomic β‐diversity in most cases. This is remarkable, given that the local environmental variability observed in the studied WTHs was very high. This highlights the strong role of spatial distance and habitat configuration in shaping community composition alongside environmental filtering (Heino, Melo, Siqueira, et al. 2015), especially in spatially discrete and ephemeral habitats such as WTHs (Córdova‐Tapia et al. 2018; Soininen 2014). Ecologically, the spatial factors likely integrate mechanisms operating from broad to fine scales in the forests studied. At broad scales, dispersal limitation and unmeasured environmental gradients, together with historical dynamics, including disturbance events across scales and isolation by geographic barriers, such as structural and geomorphological barriers that impose long‐term dispersal constraints, leave lasting imprints on communities (Dray et al. 2012). At medium to fine scales, local biotic interactions and priority effects, for example, early colonisation by dragonfly larvae suppressing other taxa and altering subsequent community composition, become important (Legendre and Legendre 2012; Padeffke and Suhling 2003; Zou and Rudolf 2023). At the same scales, life‐history traits (e.g., production of dormant stages that survive drying) and dispersal‐related traits influencing movement capacity further shape recolonization and persistence, reinforcing spatial structure (Heino, Melo, Siqueira, et al. 2015). Such patterns likely arise from trade‐offs among dispersal, dormancy, and competitive ability, which structure coexistence across space (Thompson et al. 2020; Wisnoski et al. 2019). Thus, the spatial signal most likely reflects the combined influence of environmental, biotic, and historical processes rather than dispersal limitation alone. Variation partitioning cannot definitively distinguish these processes, highlighting the need for experimental approaches to disentangle their relative importance (Smith and Lundholm 2010).

Despite our ability to explain a relatively small proportion of variation in community structure (9%–45%), this finding aligns with the typical outcomes of constrained ordination analyses based on adjusted *R* ^2^ values (Peres‐Neto et al. 2006). Low levels of explained variation are common, as demonstrated in many community studies in different ecosystems, and still allow for meaningful insights into the processes shaping metacommunities (Beisner et al. 2006; Schulz et al. 2012; Wei et al. 2025). The unexplained variation observed in our study may stem from several factors, including unaccounted environmental variables, incomplete modelling of spatial dynamics, stochastic processes or biotic interactions (Heino, Melo, Bini, et al. 2015). Although we are confident that we have accounted for most relevant predictor variables, we cannot exclude the potential additional role of these factors in affecting WTH metacommunities (Chase 2010; Leibold and Chase 2017). Given the pronounced temporal fluctuations in WTHs, stochastic processes related to habitat discovery and ecological drift likely exert substantial influence on macroinvertebrate community dynamics.

Our study highlights the important role of spatial factors for taxonomic and functional β‐diversity in WTH communities, with a greater contribution of richness differences than turnover at the taxonomic level across all studied forest types. These findings are consistent with research on other ephemeral aquatic systems, such as rock pools and temporary ponds, where frequent disturbances result in stochastic recolonization, emphasising dispersal limitations (Holyoak et al. 2020; Soininen et al. 2013). When disturbances are frequent, recolonization becomes increasingly stochastic and reliant on dispersal processes, making spatial factors a key driver of β‐diversity (Cadotte 2007; Vanschoenwinkel et al. 2013). Our analysis of functional diversity revealed biogeographical differences: in the Palaeotropical forest functional turnover prevailed over richness differences, which might reflect its long‐term environmental stability and unique evolutionary history. Integrating taxonomic and functional perspectives thus allows for deeper insights into metacommunity dynamics in temporary aquatic ecosystems and underscores the importance of spatial factors, such as dispersal constraints, for protecting fragmented, unstable habitats.

These insights have direct implications for forest management and conservation. In forests where β‐diversity is dominated by richness differences, protecting sites with high local diversity, such as large WTHs and thus old trees, should be prioritised. In contrast, where β‐diversity is mainly driven by turnover, conservation should focus on maintaining networks of multiple WTHs, as the communities in each cavity contribute distinct species or functional traits. When spatial processes dominate community assembly, conservation effectiveness also depends on safeguarding an extensive network of suitable habitats that maintains metacommunity dynamics and thus long‐term species coexistence at the regional scale. When habitat loss and isolation increase, dispersal among patches and thus connectivity may decline, increasing the risk of local and regional extinctions. Therefore, protecting large, species‐rich patches which provide source populations within extensive and well‐connected forest areas remains critical to sustain biodiversity across spatial scales.

## Author Contributions

Andreas Bruder, Martin M. Gossner and Thibaut Rota conceived the ideas and designed the methodology of the study. Francesca Cerroti and Martin M. Gossner developed the ideas for this manuscript. Francesca Cerroti and Thibaut Rota led the data collection, with the contribution of all authors. Francesca Cerroti, Thibaut Rota and Francisco Valente‐Neto identified and counted the organisms. Francesca Cerroti analysed the data. Francesca Cerroti wrote the first draft of the manuscript with the support of Martin M. Gossner. Andreas Bruder and Martin M. Gossner acquired funding and provided supervision. All authors contributed substantially to the writing of the manuscript until its final form.

## Funding

This work was funded by Schweizerischer Nationalfonds zur Förderung der Wissenschaftlichen Forschung, 315230_204998. Additional, co‐authors were supported by Fundação de Amparo à Pesquisa do Estado de São Paulo, 2019/08474‐8, 2021/13299‐0, 2022/10765‐3, 2023/01589‐0 and Anusandhan National Research Foundation (ANRF)‐Core Research Grant, CRG/2021/007839.

## Conflicts of Interest

The authors declare no conflicts of interest.

## Supporting information


**Data S1:** ele70294‐sup‐0001‐Supinfo.docx.

## Data Availability

The data and code supporting the results are available at https://zenodo.org/records/17775256, DOI: 10.5281/zenodo.17775255.
