# Supplementary material for: Spatial Factors Shape Taxonomic and Functional Beta‐Diversity in Water‐Filled Tree Holes in Different Biogeographical Regions
Source: Ecol Lett. 2025 Dec 28;28(12):e70294. doi: 10.1111/ele.70294 (PMC12744896; doi:10.1111/ele.70294)
Supplement: Supplementary file 1 — Data S1: ele70294‐sup‐0001‐Supinfo.docx. [file ELE-28-0-s001.docx]

**Supporting information**

**Title:** **Spatial factors shape taxonomic and functional beta-diversity in water-filled tree holes in different biogeographical regions**

Francesca Cerroti^1,2,3^, Thibaut Rota^1,2^, Francisco Valente-Neto^4^, K.T. Fahis^5^, Red Calore^1^, Gustavo Q. Romero^4^, Karumampoyil Sakthidas Anoop Das^5^, Andreas Bruder^1^ & Martin M. Gossner^2,3^

^1^ Institute of Microbiology, Scuola Universitaria Professionale della Svizzera Italiana (SUPSI), Via Flora Ruchat-Roncati 15, CH-6850 Mendrisio, Switzerland

^2^ Forest Entomology, Swiss Federal Institute for Forest, Snow and Landscape Research WSL, Zürcherstrasse 111, CH-8903 Birmensdorf, Switzerland

^3^ Institute of Terrestrial Ecosystems, ETH Zürich, Department of Environmental Systems Science, Rämistrasse 101, CH-8092 Zürich, Switzerland

^4^ Instituto de Biologia, Departamento de Biologia Animal, Universidade Estadual de Campinas (UNICAMP), CP 6109, CEP 13083-970 Campinas, São Paulo, Brazil

^5^ Center for Conservation Ecology & Department of Zoology, M.E.S Mampad College (Calicut University), Mampad College PO, Malappuram - 676 542, Kerala, India.

**Supporting Tables:**

**Supporting S1: Table S1*.*** Characteristics of the studied Temperate-Mediterranean forest, Neotropical rainforest, and Palaeotropical rainforest.

| **Parameter** | **Temperate-Mediterranean Massane Forest (France)** | **Neotropical**  **Amazonian Rainforest (Brazil)** | **Palaeotropical Kakkadampoyil Rainforest (India)** |
| --- | --- | --- | --- |
| **Elevation** | 700 - 1,200 m | 70 - 700 m | 650- 2,600 m |
| **Area of the Forest** | Part of 337 ha of a protected forest, designated as one site of the UNESCO world natural heritage sites “Ancient and Primeval Beech Forests of the Carpathians and other Regions of Europe” in 2021 | Part of 7000 ha of continuous forest | Located within the South Western Ghats rainforests (approximately 2,250,000 ha), is embedded in a fragmented mosaic of tropical wet evergreen forests, plantations, and agricultural lands. |
| **Climate** | Temperate - Mediterranean: 2,200 - 2,600 mm rainfall, four well-defined seasons. Winter (Dec - Feb), Spring (Mar - Jun), Summer (Jul - Sep), Autumn (Oct - Nov) | Tropical: annual temperature of 26 - 27°C (79 - 81°F) and high humidity (> 80%). Marked dry season (May to October) and rainy season (November to April) | Tropical: 24 - 27°C (75 - 80°F), rainfall from 2,200 - 4,300 mm. Two major monsoon seasons: SW (Jun - Sep) and NE (Nov - Dec), minor dry period (Jan - Apr) |
| **Forest Type** | Beech forest | Neotropical rain forest | Palaeotropical rain forest |
| **Common Trees Found** | European beech (Fagus sylvatica) with an understory dominated by European holly (Ilex aquifolium) and a low understorey with Boxwood (Buxus sempervirens) | Highly diverse. Some common families: Moraceae (e.g. Ficus), Copaifera langsdorffii (Copaíba), Bertholletia excelsa (Brazil nut), species from Meliaceae, Sapotaceae, Fabaceae, among others | High species diversity, typical evergreen broadleaf species. Includes some shrubs, herbs, epiphytes. |
| **Soil** | Dystric Cambisols: moderately acidic soils with limited base saturation. Well-drained and fertile. 2) Leptosols (Skeletic Leptosols) & Podzols (Spodic Cambisols) | Predominantly nutrient-poor soils (Ultisols, Oxisols) with low nutrient availability. Taxonomically, Ferralsols (WRB classification). Some areas are Inceptisols. | Mollisols soil (Palexhumbults). Thick, dark surface horizons, high organic matter. Well-drained but can vary in certain areas. |
| **Mean and range of water-volume per ha contained in WTHs** | Mean: 6.05 L Range 1.25-17 L | Mean: 9 L Range: 0.7 – 30.9 L | Mean: 18.93 L Range: 1.7 – 86.2 L |
| **References** | Charles et al., 2013; Mansourian et al., 2017 | Wright et al., 2011; Tovar et al., 2014 | Kettel, 2020; Panicker et al., 2018; Monsoon & Surendran, 2021 |

**Supporting S1: Table S. 2** Frequency, i.e., the number of WTHs they were present in, and total abundance of all organisms found in the tree holes in Temperate-Mediterranean forest (France), the Neotropical rainforest (Brazil), and the Palaeotropical ( India) ().

| **Temperate-Mediterranean Forest** | | | |
| --- | --- | --- | --- |
| **Family** | **Taxa** | **Frequency** | **Total Abundance** |
|  | **Diptera** |  |  |
| Ceratopogonidae | *Culicoides* | 14 | 424 |
|  | *Dasyhelea* | 16 | 159 |
| Chironomidae | *Metriocnemus cavicola* | 28 | 1089 |
|  | *Telmatogoninae* | 9 | 90 |
| Culicidae | *Aedes geniculatus* | 15 | 306 |
|  | *Anopheles plumbeus* | 3 | 68 |
| Psychodidae | *Telmatoscopini* | 8 | 17 |
| Syrphidae | *Myathropa florea* | 8 | 25 |
| Stratiomydae | *Odontomyia* | 5 | 36 |
|  | **Coleoptera** |  |  |
| Scirtidae | *Prionocyphon serricornins* | 20 | 1217 |
| **Neotropical Forest** | | | |
| **Family** | **Taxa** | **Frequency** | **Total Abundance** |
|  | **Diptera** |  |  |
| Ceratopogonidae | Ceratopogonidae | 14 | 65 |
| Culicidae | *Aedes* | 14 | 71 |
|  | *Culex* | 21 | 144 |
|  | *Culicidae* | 6 | 16 |
|  | *Orthopodomyia* | 16 | 151 |
| Empididae | Empididae | 7 | 35 |
| Psychodidae | Psychodidae | 3 | 48 |
| Syrphidae | Syrphidae | 12 | 80 |
| Stratiomydae | *Odontomyia* | 10 | 100 |
|  | **Coleoptera** |  |  |
| Dytiscidae | *Hydaticus* | 6 | 13 |
|  | *Colymbetinae* | 7 | 22 |
| Dryopidae | Dryopidae | 24 | 270 |
| Scirtidae | Scirtidae | 25 | 397 |
|  | **Hemiptera** | 5 | 6 |
|  | *Microvelia* | 5 | 6 |
|  | **Odonata** |  |  |
| Aeshnidae | *Castoraeschna* | 3 | 5 |
| Coenagrionidae | *Argia* | 8 | 10 |
|  | *Coenagrionidae* | 6 | 6 |
|  | **Others** |  |  |
| Unidentified | Nematoda | 4 | 38 |
| Unidentified | Oligochaeta | 27 | 766 |
| Unidentified | Ostracoda | 14 | 3458 |
| **Palaeotropical forest** | | | |
| **Family** | **Taxa** | **Frequency** | **Total Abundance** |
|  | **Diptera** |  |  |
| Ceratopogonidae | Ceratopogonidae_A | 7 | 41 |
| Chironomidae | Chironomidae_A | 29 | 248 |
|  | Chironomidae_B | 4 |  |
| Culicidae | Culicidae_A | 22 | 259 |
|  | Culicidae_B | 29 | 1097 |
|  | Culicidae_C | 14 | 36 |
| Muscidae | Muscidae_A | 6 | 13 |
| Psychodidae | Psycodidae | 5 | 5 |
| Unknown | Diptera_A | 4 | 5 |
|  | **Coleoptera** |  |  |
| Elmidae | Elmidae_A | 4 | 13 |
| Dytiscidae | Dytiscidae | 13 | 373 |
| Scirtidae | Scirtidae | 34 | 2390 |
|  | **Hemiptera** |  |  |
| Veliidae | Microvelia | 9 | 36 |
|  | **Others** |  |  |
| Unidentified | Acaria | 17 | 39 |
| Unidentified | Anura larvae | 3 | 22 |
| Unidentified | Nematoda | 3 | 7 |
| Unidentified | Oligochaeta | 29 | 760 |
| Unidentified | Ostracoda | 34 | 17653 |
| Unidentified | Bosmina | 27 | 2909 |
| Unidentified | Copepoda | 33 | 5074 |
| Gecarcinucidae | Crab | 12 | 24 |

**Supporting S1: Table S. 3**Mean, Standard Deviation (SD) and coefficient of variation (CV) of local environmental variables measured in tree holes of the different forests.

|  | **Site** | | | | | | | | |
| --- | --- | --- | --- | --- | --- | --- | --- | --- | --- |
|  | **Temperate-Mediterranean** | | | **Neotropical** | | | **Palaeotropical** | | |
| **Variable** | **Mean** | **SD** | **CV** | **Mean** | **SD** | **CV** | **Mean** | **SD** | **CV** |
| Ammonium (mg/L) | 0.88 | 1.00 | 113.64 | NA | NA | NA | 7.19 | 0.71 | 9.84 |
| Chlorid (mg/L) | 39.23 | 33.49 | 131.99 | 9.25 | 12.21 | 131.99 | NA | NA | NA |
| CDOM (μg/L) | NA | NA | NA | 222.10 | 181.85 | 81.88 | 126.20 | 49.59 | 39.30 |
| Chlorophill A (μg/L) | NA | NA | NA | 84.67 | 64.70 | 76.42 | 47.73 | 100.51 | 210.57 |
| Circumference (cm) | 132.56 | 71.47 | 53.92 | 97.06 | 46.54 | 47.95 | 149.41 | 121.68 | 81.44 |
| Coarse organic matter (g) | 72.93 | 86.72 | 118.91 | 80.33 | 75.80 | 94.36 | 39.70 | 70.38 | 177.27 |
| Conductivity (μS/cm) | 947.39 | 1872.81 | 197.68 | 2156.41 | 6649.72 | 308.37 | 128.34 | 139.72 | 108.86 |
| Fine organic matter - 300 (g) | 20.56 | 30.29 | 147.29 | 24.08 | 29.23 | 121.35 | 13.85 | 26.48 | 191.14 |
| Fine organic matter - 500 (g) | 41.32 | 56.58 | 136.94 | 18.97 | 21.30 | 112.28 | 17.83 | 43.42 | 243.49 |
| Height (cm) | 128.64 | 160.84 | 125.03 | 577.84 | 366.86 | 63.49 | 67.26 | 89.18 | 132.58 |
| Nitrate (mg/L) | 1.46 | 3.39 | 233.04 | 3.42 | 4.54 | 132.75 | NA | NA | NA |
| Nitrite (mg/L) | 0.04 | 0.04 | 109.67 | 0.07 | 0.11 | 164.18 | 18.51 | 10.34 | 55.84 |
| Opening area (cm²) | 214.46 | 161.80 | 75.45 | 171.76 | 136.12 | 79.25 | 216.60 | 199.14 | 91.94 |
| Oxygen (mg/L) | 3.55 | 3.12 | 87.89 | 1.80 | 0.94 | 52.48 | 1.45 | 1.89 | 130.07 |
| pH | 7.04 | 0.73 | 10.40 | 7.36 | 1.57 | 21.36 | 6.35 | 0.38 | 6.01 |
| Phosphate (mg/L) | 14.77 | 63.93 | 432.73 | 7.44 | 11.78 | 158.33 | 21.50 | 3.87 | 18.00 |
| Saturation % | 34.87 | 29.44 | 84.42 | 3.56 | 2.23 | 62.60 | 14.27 | 9.39 | 65.79 |
| Sulfate (mg/L) | 3.38 | 4.65 | 137.30 | 2.44 | 5.60 | 229.51 | NA | NA | NA |
| Turbidity (FNU) | NA | NA | NA | 66.08 | 111.00 | 167.96 | 27.31 | 19.22 | 70.37 |
| Water temp. (°C) | 10.90 | 2.37 | 21.71 | 24.23 | 1.26 | 5.21 | 23.13 | 0.34 | 1.48 |
| Water volume (L) | 3.06 | 2.19 | 71.45 | 2.37 | 2.63 | 111.14 | 4.70 | 9.45 | 201.05 |

**Supporting S1: Table S. 4 a.** Results of spearman correlations analysis among predictors for the Temperate-Mediterranean forest.

| Temperate-Mediterranean forest | Coarse organic matter (g) | Fine organic matter (500) | Fine organic matter (300) | Orien. | pH | Oxygen (mg/L) | Oxygen  Sat.(%) | Cond.  (μg/L) | Water Vol (L) | Coarse organic matter (g) | Height (cm) | Amm. (mg/L) | Chlorid (mg/L) | Nitrit (mg/L) | Nitrat (mg/L) | Phosphat (mg/L) | Sulfat (mg/L) |
| --- | --- | --- | --- | --- | --- | --- | --- | --- | --- | --- | --- | --- | --- | --- | --- | --- | --- |
| Coarse organic matter (g) | 1 |  |  |  |  |  |  |  |  |  |  |  |  |  |  |  |  |
| Fine organic matter - 500 (g) | 0.68 | 1 |  |  |  |  |  |  |  |  |  |  |  |  |  |  |  |
| Fine organic matter - 300 (g) | 0.62 | 0.85 | 1 |  |  |  |  |  |  |  |  |  |  |  |  |  |  |
| Orientation | -0.14 | -0.08 | 0.04 | 1 |  |  |  |  |  |  |  |  |  |  |  |  |  |
| pH | -0.22 | -0.01 | 0.03 | 0.03 | 1 |  |  |  |  |  |  |  |  |  |  |  |  |
| Oxygen (mg/L) | -0.27 | 0 | 0.06 | 0.22 | -0.21 | 1 |  |  |  |  |  |  |  |  |  |  |  |
| Oxygen Saturation (%) | -0.27 | -0.01 | 0.05 | 0.25 | -0.25 | 0.98 | 1 |  |  |  |  |  |  |  |  |  |  |
| Conductivity (μS/cm) | -0.16 | -0.18 | -0.15 | -0.08 | 0.74 | -0.35 | -0.38 | 1 |  |  |  |  |  |  |  |  |  |
| Water Volume (L) | 0.69 | 0.49 | 0.45 | -0.24 | -0.02 | -0.21 | -0.28 | 0.1 | 1 |  |  |  |  |  |  |  |  |
| Circumference (cm) | 0.42 | 0.32 | 0.12 | 0.08 | -0.1 | -0.21 | -0.19 | -0.25 | 0.19 | 1 |  |  |  |  |  |  |  |
| Height (cm) | -0.28 | -0.41 | -0.17 | -0.23 | 0.34 | -0.14 | -0.17 | 0.45 | 0.01 | -0.42 | 1 |  |  |  |  |  |  |
| Ammonium (mg/L) | 0.05 | -0.04 | 0.05 | -0.26 | -0.14 | -0.29 | -0.26 | 0 | 0.13 | -0.15 | 0.38 | 1 |  |  |  |  |  |
| Chlorid (mg/L) | 0.04 | -0.11 | -0.05 | 0.06 | -0.11 | -0.04 | -0.04 | 0.12 | 0.15 | 0 | 0.3 | 0.54 | 1 |  |  |  |  |
| Nitrite (mg/L) | 0.14 | 0.01 | 0.02 | -0.26 | -0.26 | -0.06 | -0.04 | -0.32 | -0.06 | 0.13 | -0.12 | 0.14 | -0.01 | 1 |  |  |  |
| Nitrate (mg/L) | -0.03 | -0.16 | -0.17 | 0.08 | -0.26 | 0.23 | 0.24 | -0.25 | -0.36 | 0.04 | -0.23 | -0.25 | 0.08 | 0.29 | 1 |  |  |
| Phosphate (mg/L) | -0.11 | -0.07 | -0.05 | -0.17 | 0.74 | -0.31 | -0.37 | 0.71 | 0.22 | -0.14 | 0.41 | -0.03 | 0.15 | -0.32 | -0.27 | 1 |  |
| Sulfat (mg/L) | -0.3 | -0.38 | -0.26 | 0.06 | -0.26 | 0.2 | 0.17 | 0.08 | 0 | -0.26 | 0.22 | 0.11 | 0.52 | -0.03 | 0.18 | -0.09 | 1 |
| Opening area (cm²) | 0.6 | 0.36 | 0.39 | -0.01 | -0.26 | -0.12 | -0.11 | -0.1 | 0.57 | 0.22 | -0.09 | 0.29 | 0.25 | -0.09 | -0.22 | -0.2 | 0.06 |

**Supporting S1: Table S. 4.b.** Results of spearman correlations analysis among predictors for the Neotropical forest.

| Brazil | Coarse organic matter (g) | Fine organic matter (500) | Fine organic matter (300) | Orien. | pH | Oxygen (mg/L) | Oxygen  Sat.(%) | Cond.  (μg/L) | Water Vol (L) | Coarse organic matter (g) | Height (cm) | Chlorid (mg/L) | Nitrit (mg/L) | Nitrat (mg/L) | Phosp (mg/L) | Sulfat (mg/L) | Turb. (FUN) | CDOM  (μg/L) | Chloro  (μg/L). |
| --- | --- | --- | --- | --- | --- | --- | --- | --- | --- | --- | --- | --- | --- | --- | --- | --- | --- | --- | --- |
| Coarse organic matter (g) | 1 |  |  |  |  |  |  |  |  |  |  |  |  |  |  |  |  |  |  |
| Fine organic matter - 500 (g) | 0.62 | 1 |  |  |  |  |  |  |  |  |  |  |  |  |  |  |  |  |  |
| Fine organic matter - 300 (g) | 0.6 | 0.74 | 1 |  |  |  |  |  |  |  |  |  |  |  |  |  |  |  |  |
| Orientation | -0.06 | -0.05 | -0.15 | 1 |  |  |  |  |  |  |  |  |  |  |  |  |  |  |  |
| pH | 0.4 | 0.43 | 0.39 | -0.12 | 1 |  |  |  |  |  |  |  |  |  |  |  |  |  |  |
| Oxygen (mg/L) | -0.2 | -0.18 | -0.02 | -0.32 | 0.07 | 1 |  |  |  |  |  |  |  |  |  |  |  |  |  |
| Oxygen Saturation (%) | -0.18 | -0.11 | 0.06 | -0.36 | 0.19 | 0.91 | 1 |  |  |  |  |  |  |  |  |  |  |  |  |
| Conductivity (μS/cm) | 0.22 | 0.2 | -0.02 | 0.31 | -0.03 | -0.47 | -0.5 | 1 |  |  |  |  |  |  |  |  |  |  |  |
| Water Volume (L) | 0.7 | 0.52 | 0.38 | -0.06 | 0.22 | -0.01 | -0.02 | 0.25 | 1 |  |  |  |  |  |  |  |  |  |  |
| Circumference (cm) | 0.41 | 0.13 | 0.04 | -0.12 | 0.1 | -0.09 | -0.04 | 0.15 | 0.41 | 1 |  |  |  |  |  |  |  |  |  |
| Height (cm) | 0.41 | -0.17 | -0.15 | 0.21 | -0.09 | -0.08 | -0.23 | 0.33 | 0.44 | 0.59 | 1 |  |  |  |  |  |  |  |  |
| Chlorid (mg/L) | -0.36 | -0.32 | -0.48 | 0.18 | -0.25 | 0.14 | 0.1 | 0.23 | -0.06 | 0.13 | 0.21 | 1 |  |  |  |  |  |  |  |
| Nitrite (mg/L) | -0.07 | 0.09 | 0.09 | 0.15 | 0.06 | -0.09 | -0.12 | 0.07 | -0.16 | 0.1 | 0.14 | 0 | 1 |  |  |  |  |  |  |
| Nitrate (mg/L) | -0.17 | -0.32 | -0.32 | 0.25 | -0.24 | -0.25 | -0.24 | 0.22 | -0.29 | 0.29 | 0.28 | 0.4 | 0.37 | 1 |  |  |  |  |  |
| Phosphate (mg/L) | 0 | -0.04 | -0.3 | 0.36 | -0.16 | -0.42 | -0.42 | 0.42 | 0.18 | 0.11 | 0.29 | 0.11 | 0.04 | 0.03 | 1 |  |  |  |  |
| Sulfat (mg/L) | 0.03 | -0.06 | -0.18 | 0.34 | -0.19 | -0.17 | -0.13 | 0.45 | 0.02 | 0.13 | 0.23 | 0.46 | 0.41 | 0.64 | 0.27 | 1 |  |  |  |
| Turb. (FUN) | 0.02 | 0.34 | 0.14 | 0.05 | 0.25 | -0.08 | -0.07 | 0.21 | 0.14 | 0.3 | 0.05 | 0.24 | 0.44 | 0.17 | 0.21 | 0.33 | 1 |  |  |
| CDOM (μg/L) | -0.12 | -0.15 | 0.24 | -0.12 | -0.1 | 0.14 | 0.11 | -0.34 | -0.17 | -0.02 | -0.06 | -0.15 | -0.11 | 0.01 | -0.4 | -0.38 | -0.1 | 1 |  |
| Chlorophill A (μg/L | -0.01 | 0.05 | 0.05 | 0.3 | -0.12 | -0.07 | -0.19 | 0.07 | 0.07 | 0.04 | 0.13 | -0.11 | 0.11 | 0.07 | 0.15 | 0.11 | 0.41 | 0.42 | 1 |
| Opening area (cm²) | 0.52 | 0.53 | 0.51 | -0.19 | 0.32 | -0.11 | 0 | 0.09 | 0.63 | 0.03 | -0.03 | -0.31 | -0.12 | -0.54 | 0.17 | -0.17 | -0.01 | -0.04 | -0.06 |

| India | Coarse organic matter (g) | Fine organic matter (500) | Fine organic matter (300) | Orien. | pH | Oxygen (mg/L) | Sat.(%) | Cond.  (μg/L) | Water Vol (L) | Circ. | Height (cm) | Ammo.(mg/L) | Nitrit (mg/L) | Phosp (mg/L) | Turbidity (FUN) | CDOM  (μg/L) | Chloro  (μg/L). |
| --- | --- | --- | --- | --- | --- | --- | --- | --- | --- | --- | --- | --- | --- | --- | --- | --- | --- |
| Coarse organic matter (g) | 1 |  |  |  |  |  |  |  |  |  |  |  |  |  |  |  |  |
| Fine organic matter - 500 (g) | 0.82 | 1 |  |  |  |  |  |  |  |  |  |  |  |  |  |  |  |
| Fine organic matter - 300 (g) | 0.66 | 0.74 | 1 |  |  |  |  |  |  |  |  |  |  |  |  |  |  |
| Orientation | -0.15 | -0.03 | 0.01 | 1 |  |  |  |  |  |  |  |  |  |  |  |  |  |
| pH | 0.08 | 0.11 | 0.13 | -0.04 | 1 |  |  |  |  |  |  |  |  |  |  |  |  |
| Oxygen (mg/L) | -0.26 | -0.29 | -0.09 | 0.18 | -0.05 | 1 |  |  |  |  |  |  |  |  |  |  |  |
| Oxygen  Saturation (%) | -0.06 | -0.08 | 0.03 | 0.01 | -0.21 | 0.26 | 1 |  |  |  |  |  |  |  |  |  |  |
| Conductivity (μS/cm) | -0.12 | -0.12 | -0.05 | -0.15 | 0.68 | -0.09 | -0.14 | 1 |  |  |  |  |  |  |  |  |  |
| Water Volume (L) | 0.85 | 0.71 | 0.64 | -0.26 | 0.05 | -0.14 | -0.01 | -0.03 | 1 |  |  |  |  |  |  |  |  |
| Circumference (cm) | 0.16 | 0.16 | 0.1 | -0.05 | 0.02 | 0.04 | 0.27 | 0.36 | 0.23 | 1 |  |  |  |  |  |  |  |
| Height (cm) | 0.02 | 0.1 | 0.1 | 0.09 | -0.18 | 0 | -0.06 | -0.21 | 0.22 | -0.11 | 1 |  |  |  |  |  |  |
| Ammo.(mg/L) | -0.02 | -0.23 | -0.14 | -0.12 | 0.2 | 0.01 | 0.2 | 0.17 | 0.12 | 0.25 | -0.06 | 1 |  |  |  |  |  |
| Nitrit (mg/L) | -0.18 | -0.24 | -0.28 | 0.02 | 0.05 | 0 | -0.04 | -0.09 | -0.22 | -0.04 | 0.08 | 0.47 | 1 |  |  |  |  |
| Phosphat (mg/L) | -0.07 | -0.11 | -0.13 | -0.13 | -0.26 | 0.24 | 0.15 | -0.22 | -0.07 | -0.13 | -0.17 | -0.12 | 0.02 | 1 |  |  |  |
| Turbidity (FUN) | -0.19 | -0.09 | -0.24 | -0.2 | 0.31 | -0.04 | -0.21 | 0.19 | -0.18 | 0.08 | 0.14 | 0.07 | 0.28 | -0.27 | 1 |  |  |
| CDOM (μg/L) | 0.03 | 0.04 | 0.06 | -0.13 | 0.57 | -0.19 | -0.15 | 0.59 | 0.01 | 0.25 | 0.14 | 0.26 | 0.2 | -0.32 | 0.29 | 1 |  |
| Chlorophill A (μg/L) | -0.11 | -0.08 | -0.13 | -0.35 | 0.42 | -0.06 | -0.09 | 0.46 | -0.08 | 0.39 | 0.03 | 0.31 | 0.36 | -0.17 | 0.67 | 0.68 | 1 |
| Opening area (cm²) | 0.69 | 0.48 | 0.39 | -0.36 | 0.18 | -0.19 | 0 | 0.08 | 0.75 | 0.2 | 0.07 | 0.33 | -0.02 | -0.17 | 0.06 | 0.15 | 0.17 |

**Supporting S1: Table S. 4.c.** Results of spearman correlations analysis among predictors for the Palaeotropical forest

**Supporting S1: Table S. 5** Environmental and spatial variables selected by the forward selection procedure for taxonomic β-diversity (and its components), for the Temperate-Mediterranean, Neotropical and, Palaeotropical forests.

| **Taxonomic** | | | | | | |
| --- | --- | --- | --- | --- | --- | --- |
| **Temperate-Mediterranean** | | | | | | |
| **Variables** | **Richness differences** | | **Species turnover** | | **β-diversity** | |
|  | **R^2^ - Adjusted** | **p-value** | **R^2^ - Adjusted** | **p-value** | **R^2^ - Adjusted** | **p-value** |
| **Environmentals** |  |  |  |  |  |  |
| Ammonium | 0.12 | 0.05 |  |  | 0.09 | 0.002 |
| Chlorid |  |  | 0.08 | 0.02 |  |  |
| Coarse organic matter(g) |  |  |  |  | 0.06 | 0.002 |
| Fine organic matter (g) | 0.06 | 0.02 |  |  |  |  |
| Height (cm) |  |  |  |  |  |  |
| Ph |  |  |  |  | 0.04 | 0.004 |
| Phosphate |  |  | 0.06 | 0.002 |  |  |
|  |  |  |  |  |  |  |
| **Spatial** |  |  |  |  |  |  |
| MEM1 | 0.06 | 0.02 | 0.07 | 0.02 | 0.03 | 0.004 |
| MEM3 |  |  |  |  |  |  |
| MEM6 |  |  | 0.04 | 0.004 | 0.06 | 0.002 |
| MEM9 |  |  | 0.1 | 0.05 |  |  |
| MEM10 |  |  | 0.12 | 0.04 |  |  |
| MEM14 |  |  | 0.09 | 0.05 | 0.08 | 0.01 |
| MEM16 |  |  |  |  | 0.1 | 0.05 |
| MEM20 | 0.09 | 0.012 |  |  |  |  |
|  |  |  |  |  |  |  |
| **Neotropical** | | | | | | |
| **Variables** | **Richness differences** | | **Species turnover** | | **β-diversity** | |
|  | **R^2^ - Adjusted** | **p-value** | **R^2^ - Adjusted** | **p-value** | **R^2^ - Adjusted** | **p-value** |
| **Environmentals** |  |  |  |  |  |  |
| Coarse organic matter (g) | 0.12 | 0.002 |  |  | 0.03 | 0.008 |
| Fine organic matter (g) |  |  | 0.03 | 0.02 |  |  |
|  |  |  |  |  |  |  |
| **Spatial** |  |  |  |  |  |  |
| MEM7 |  |  | 0.02 | 0.05 |  |  |
| MEM15 | 0.19 | 0.05 |  |  |  |  |
| MEM17 | 0.14 | 0.004 |  |  | 0.03 | 0.004 |
| MEM18 |  |  |  |  |  |  |
| MEM19 |  |  |  |  | 0.04 | 0.04 |
| MEM26 |  |  | 0.04 | 0.04 |  |  |
| MEM27 | 0.23 | 0.048 |  |  |  |  |
| MEM30 |  |  | 0.02 | 0.04 |  |  |
|  |  |  |  |  |  |  |
| **Palaeotropical** | | | | | | |
| **Variables** | **Richness differences** | | **Species turnover** |  | **β-diversity** |  |
|  | **R^2^ - Adjusted** | **p-value** | **R^2^ - Adjusted** | **p-value** | **R^2^ - Adjusted** | **p-value** |
| **Environmentals** |  |  |  |  |  |  |
| Ammonium | 0.15 | 0.028 |  |  |  |  |
| Ph |  |  | 0.05 | 0.05 |  |  |
| Turbidity | 0.08 | 0.03 |  |  |  |  |
| Orientation |  |  | 0.03 | 0.012 |  |  |
| **Spatial** |  |  |  |  |  |  |
| MEM1 |  |  | 0.02 | 0.03 |  |  |
| MEM4 | 0.13 | 0.02 |  |  | 0.03 | 0.022 |
| MEM12 | 0.21 | 0.03 |  |  |  |  |
| MEM21 |  |  | 0.02 | 0.05 |  |  |
| MEM26 |  |  |  |  | 0.02 | 0.04 |

**Supporting S1: Table S. 6** Environmental and spatial variables selected by the forward selection procedure for functional β-diversity (and its components), for the Temperate-Mediterranean, Neotropical and, Palaeotropical forests.

| **Functional** | | | | | | |
| --- | --- | --- | --- | --- | --- | --- |
| **Temperate-Mediterranean** | | | | | | |
| **Variables** | **Richness differences** | | **Functional turnover** | | **β-diversity** | |
|  | **R^2^ - Adjusted** | **p-value** | **R^2^ - Adjusted** | **p-value** | **R^2^ - Adjusted** | **p-value** |
| **Environmentals** |  |  |  |  |  |  |
| Ammonium |  |  | 0.06 | 0.01 |  |  |
|  |  |  |  |  |  |  |
| Chlorid |  |  |  |  | 0.06 | 0.012 |
|  |  |  |  |  |  |  |
| Spatial |  |  |  |  |  |  |
| MEM1 |  |  | 0.11 | 0.002 | 0.07 | 0.02 |
| MEM21 |  |  | 0.07 | 0.04 |  |  |
| MEM19 | 0.15 | 0.04 |  |  |  |  |
| MEM30 |  |  |  |  | 0.04 | 0.02 |
| MEM33 | 0.08 | 0.03 |  |  |  |  |
| MEM24 | 0.21 | 0.04 |  |  |  |  |
|  |  |  |  |  |  |  |
|  |  | **Neotropical** | |  |  |  |
| **Variables** | **Richness difference** | | **Functional turnover** |  | **β-diversity** |  |
|  | **R^2^ - Adjusted** | **p-value** | **R^2^ - Adjusted** | **p-value** | **R^2^ - Adjusted** | **p-value** |
| **Environmentals** |  |  |  |  |  |  |
| Oxigen (mg/L) | 0.12 | 0.03 |  |  | 0.04 | 0.04 |
| Nitrit |  |  |  |  |  |  |
| **Spatial** |  |  |  |  |  |  |
| MEM1 |  |  |  |  |  |  |
| MEM14 |  |  | 0.08 | 0.01 |  |  |
| MEM16 | 0.14 | 0.01 |  |  | 0.07 | 0.006 |
| MEM19 | 0.21 | 0.05 |  |  |  |  |
| MEM32 |  |  | 0.02 | 0.05 |  |  |
|  |  |  |  |  |  |  |
| **Palaeotropical** | | | | | | |
| **Variables** | **Richness difference** | | **Functional turnover** |  | **β-diversity** |  |
|  | **R^2^ - Adjusted** | **p-value** | **R^2^ - Adjusted** | **p-value** | **R^2^ - Adjusted** | **p-value** |
| **Environmentals** |  |  |  |  |  |  |
| Turbidity | 0.11 | 0.03 |  |  |  |  |
| Height |  |  |  |  |  |  |
| pH |  |  | 0.05 | 0.09 | 0.06 | 0.032 |
| **MEM** |  |  |  |  |  |  |
| MEM1 |  |  | 0.08 | 0.004 | 0.12 | 0.002 |
| MEM10 |  |  | 0.1 | 0.05 |  |  |
| MEM19 | 0.35 | 0.016 |  |  |  |  |
| MEM21 | 0.43 | 0.016 |  |  | 0.04 | 0.05 |
| MEM24 | 0.17 | 0.01 |  |  |  |  |
| MEM29 |  |  |  |  |  |  |
| MEM33 | 0.26 | 0.03 | 0.04 | 0.05 |  |  |

**Supporting S1: Table S. 7** Variation partitioning analysis for taxonomic β-diversity (and its components) for the Temperate-Mediterranean, Neotropical and, Palaeotropical forests. Significance is indicated at P <0.05 (in bold). E=environmental variation, S= spatial variation, E+S= Total explained variation, E|S= Pure environmental variation, S|E= Pure spatial variation, b= Variation shared by environmental and spatial factors, R= Unexplained variation (residuals).

| **Taxonomic** | | | | | | |
| --- | --- | --- | --- | --- | --- | --- |
| **Temperate-Mediterranean** | **Richness differences** | | **Species turnover** | | **β-diversity** | |
|  | **R² adjusted** | **p-value** | **R² adjusted** | **p-value** | **R² adjusted** | **p-value** |
| E | 0.12 | **0.01** | 0.08 | **0.001** | 0.08 | **0.001** |
| S | 0.15 | **0.009** | 0.12 | **0.001** | 0.09 | **0.001** |
| E+S | 0.2 | **0.07** | 0.14 | 0.08 | 0.15 | **0.002** |
|  |  |  |  |  |  |  |
| E\|S | 0.05 | 0.06 | 0.02 | 0.08 | 0.06 | **0.001** |
| S\|E | 0.08 | **0.031** | 0.06 | **0.004** | 0.06 | **0.002** |
| b | 0.07 | NA | 0.06 | NA | 0.03 | NA |
| R | 0.79 | NA | 0.86 | NA | 0.85 | NA |
|  |  |  |  |  |  |  |
| **Neotropical** | **Richness differences** | | **Species turnover** | | **β-diversity** | |
|  | **R² adjusted** | **p-value** | **R² adjusted** | **p-value** | **R² adjusted** | **p-value** |
| E | 0.12 | **0.006** | 0.03 | **0.01** | 0.03 | **0.006** |
| S | 0.23 | **0.001** | 0.06 | **0.005** | 0.05 | **0.003** |
| E+S | 0.23 | 0.28 | 0.08 | **0.026** | 0.07 | **0.01** |
|  |  |  |  |  |  |  |
| E\|S | 0.005 | 0.28 | 0.02 | **0.02** | 0.02 | **0.01** |
| S\|E | 0.11 | **0.02** | 0.05 | **0.005** | 0.04 | **0.004** |
| b | 0.12 | NA | 0.004 | NA | 0.01 | NA |
| R | 0.76 | NA | 0.91 | NA | 0.93 | NA |
|  |  |  |  |  |  |  |
| **Palaeotropical** | **Richness differences** | | **Species turnover** | | **β-diversity** | |
|  | **R² adjusted** | **p-value** | **R² adjusted** | **p-value** | **R² adjusted** | **p-value** |
| E | 0.15 | **0.014** | 0.05 | **0.004** | 0.01 | 0.08 |
| S | 0.21 | **0.003** | 0.04 | **0.009** | 0.03 | **0.04** |
| E+S | 0.31 | **0.03** | 0.08 | **0.007** | 0.06 | **0.026** |
|  |  |  |  |  |  |  |
| E\|S | 0.1 | **0.03** | 0.04 | **0.02** | 0.03 | **0.026** |
| S\|E | 0.16 | **0.008** | 0.03 | **0.05** | 0.04 | **0.013** |
| b | 0.05 | NA | 0.01 | NA | -0.01 | NA |
| R | 0.69 | NA | 0.91 | NA | 0.94 | NA |

**Supporting S1: Table S. 8** Variation partitioning analysis for functional β diversity (and its components) for the Temperate-Mediterranean, Neotropical and, Palaeotropical forests, significant values P <0.05 (in bold). E=environmental variation, S= spatial variation, E+S= Total explained variation, E|S= Pure environmental variation, S|E= Pure spatial variation, b= Variation shared by environmental and spatial factors, R= Unexplained variation (residuals)

| **Functional** | | | | | | |
| --- | --- | --- | --- | --- | --- | --- |
| **Temperate-Mediterranean** | **Richness differences** | | **Functional turnover** | | **β-diversity** | |
|  | **R² adjusted** | **p-value** | **R² adjusted** | **p-value** | **R² adjusted** | **p-value** |
| E | 0.08 | **0.03** | 0.07 | **0.007** | 0.06 | **0.006** |
| S | 0.2 | **0.001** | 0.2 | **0.001** | 0.12 | **0.001** |
| E+S | 0.29 | **0.014** | 0.2 | 0.43 | 0.15 | **0.003** |
|  |  |  |  |  |  |  |
| E\|S | 0.08 | **0.01** | -0.001 | 0.44 | 0.03 | **0.05** |
| S\|E | 0.21 | **0.001** | 0.13 | **0.002** | 0.09 | **0.004** |
| b | 0.0008 | NA | 0.07 | NA | 0.03 | NA |
| R | 0.71 | NA | 0.80 | NA | 0.85 | NA |
|  |  |  |  |  |  |  |
| **Neotropical** | **Richness differences** | | **Functional turnover** | | **β-diversity** | |
|  | **R² adjusted** | **p-value** | **R² adjusted** | **p-value** | **R² adjusted** | **p-value** |
| E | 0.12 | **0.03** | 0.02 | 0.134 | 0.04 | **0.05** |
| S | 0.21 | **0.01** | 0.12 | **0.004** | 0.07 | **0.007** |
| E+S | 0.31 | **0.02** | 0.14 | 0.11 | 0.08 | **0.04** |
|  |  |  |  |  |  |  |
| E\|S | 0.1 | **0.02** | 0.02 | 0.107 | 0.01 | 0.2 |
| S\|E | 0.19 | **0.01** | 0.11 | **0.004** | 0.04 | **0.03** |
| b | 0.02 | NA | 0.003 | NA | 0.03 | NA |
| R | 0.69 | NA | 0.85 | NA | 0.91 | NA |
|  |  |  |  |  |  |  |
| **Palaeotropical** | **Richness differences** | | **Functional turnover** | | **β-diversity** | |
|  | **R² adjusted** | **p-value** | **R² adjusted** | **p-value** | **R² adjusted** | **p-value** |
| E | 0.11 | **0.032** | 0.06 | **0.03** | 0.06 | **0.02** |
| S | 0.42 | **0.001** | 0.22 | **0.002** | 0.2 | **0.002** |
| E+S | 0.45 | 0.11 | 0.20 | 0.9 | 0.16 | **0.005** |
|  |  |  |  |  |  |  |
| E\|S | 0.03 | 0.1 | -0.02 | 0.9 | -0.005 | 0.52 |
| S\|E | 0.34 | **0.001** | 0.14 | **0.007** | 0.1 | **0.004** |
| b | 0.08 | NA | 0.07 | NA | 0.07 | NA |
| R | 0.55 | NA | 0.79 | NA | 0.83 | NA |

**Supporting S1:** **Figure S1 Mean and recent 5-day precipitation patterns across the studied biogeographic regions.**


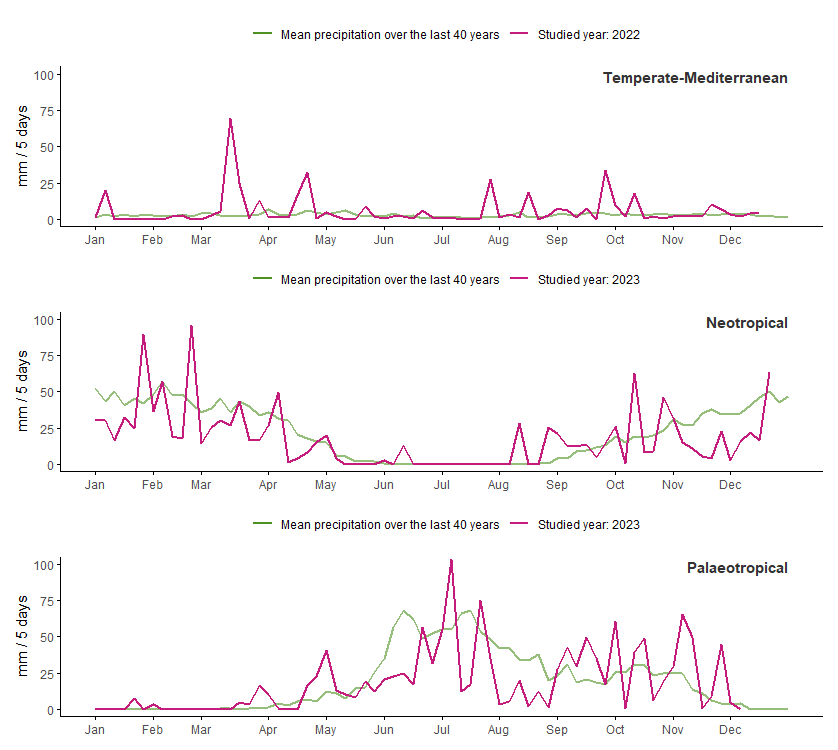

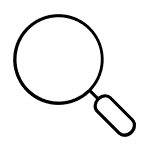

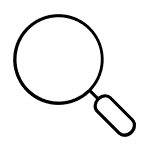

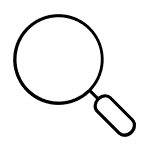


**Figure S1.** Temporal evolution of precipitation (mm per 5 days) throughout the year for the three studied biogeographical regions: Temperate-Mediterranean, Neotropical, and Palaeotropical. The green line represents mean precipitation summed over 5-day periods (pentads) averaged across the last 40 years, while the magenta line shows 5-day precipitation totals for the most recent year of observation (2022-2023, depending on the region). Daily precipitation data were obtained from the NASA POWER database and aggregated into consecutive 5-day periods. February 29th was excluded to maintain a consistent 365-day calendar year. The magnifying glass marks the sampling period.

**Supporting S1: Figure S2 Schematic representation of spatial structures captured by Moran’s Eigenvector Maps (MEMs) at multiple spatial scales.**


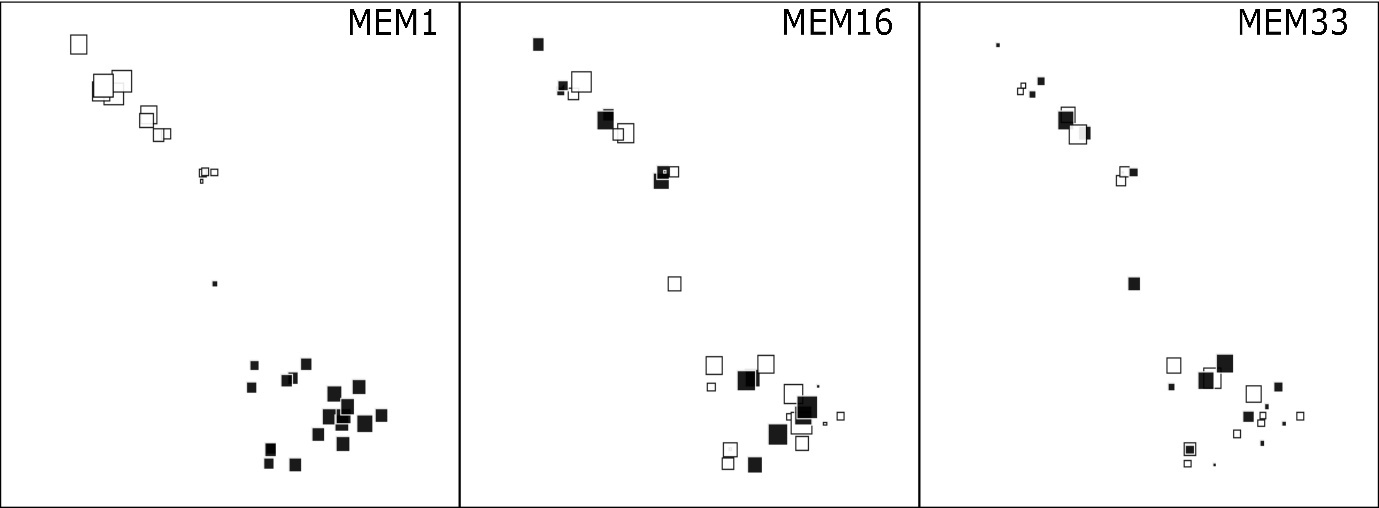


**Figure S2.** Each panel illustrates a different MEM variable, revealing spatial autocorrelation patterns in the dataset. The horizontal and vertical axes correspond to geographic coordinates (longitude and latitude, respectively). Each square represents a sampled WTH, with its size indicating the absolute value of the corresponding MEM eigenvector. Black squares denote positive values, while white squares indicate negative values. The MEMs exhibit a wave-like spatial pattern: broad-scale MEMs (e.g., MEM1) show high-amplitude, low-frequency variations, capturing large-scale spatial gradients; medium-scale MEMs (e.g., MEM12) reflect intermediate spatial structures; and fine-scale MEMs (e.g., MEM33) display low-amplitude, high-frequency patterns, indicating more localized variations among neighbouring sites.

**
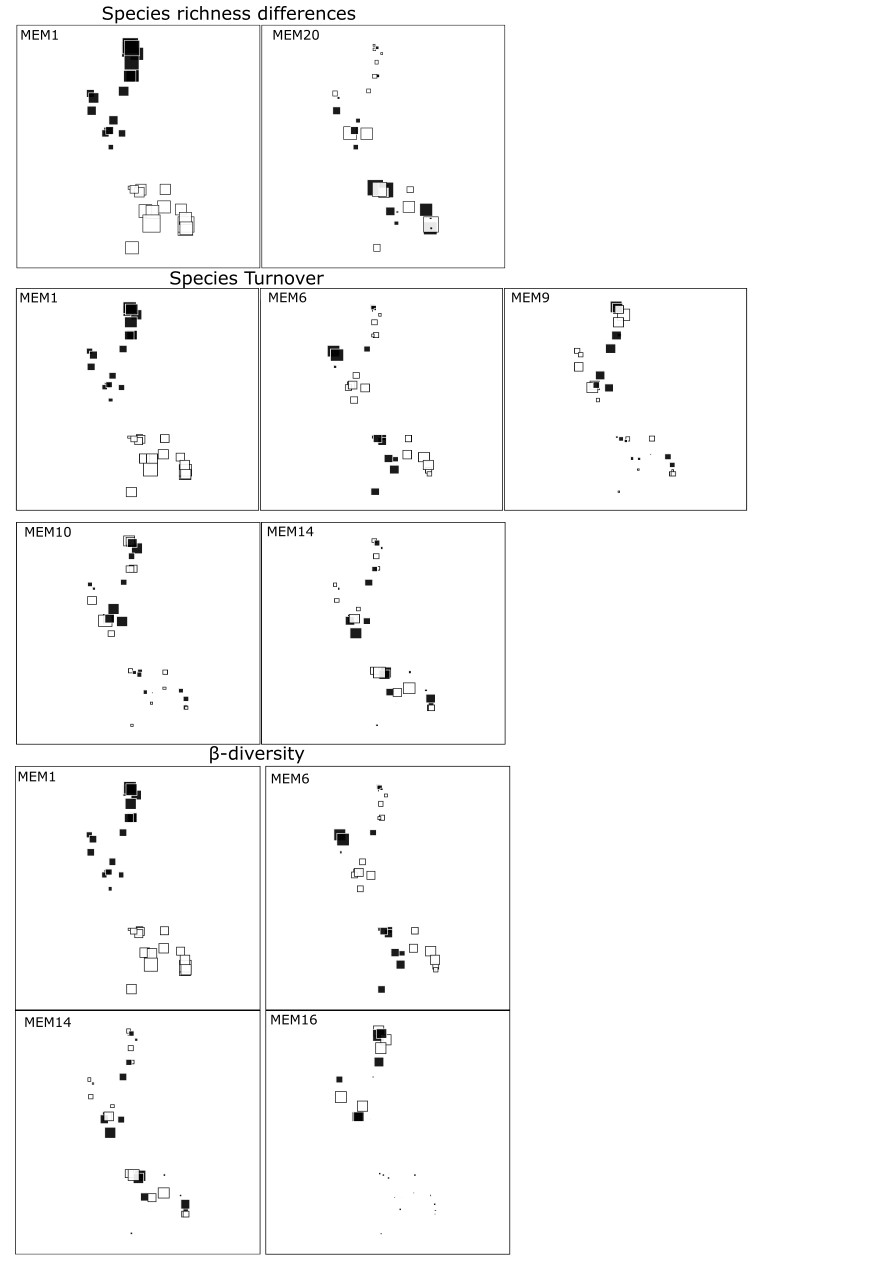
Supporting S1: Figure S3** Schematic representation of the spatial variables selected by the forward selection procedure for the Temperate-Mediterranean forest taxonomic β-diversity and its components.

Broad scale

Medium scaleoad scale scale

Medium scale

Medium scaleoad scale scale

Broad scale

Broad scale

Medium scale scale scale

Broad scale

Broad scale

Broad scale

Broad scale


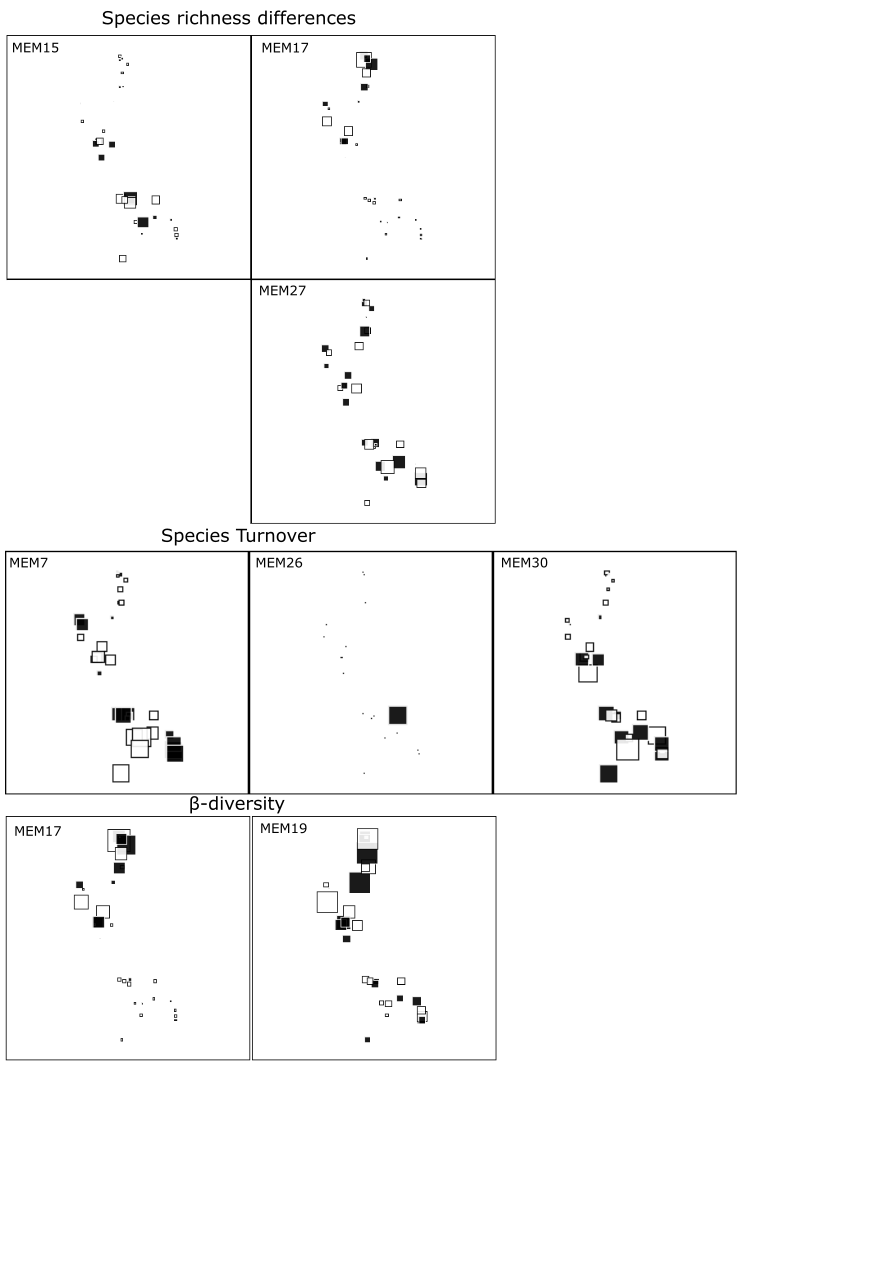
**Supporting S1: Figure S4** Schematic representation of the spatial variables selected by the forward selection procedure for the Neotropical forest taxonomic β-diversity and its components.

Medium scaleoad scale scale

Medium scaleoad scale scale

Broad scaleoad scale scale

Fine scaleoad scale scale

Medium scaleoad scale scale

Fine scaleoad scale scale

Medium scaleoad scale scale

Medium scaleoad scale scale


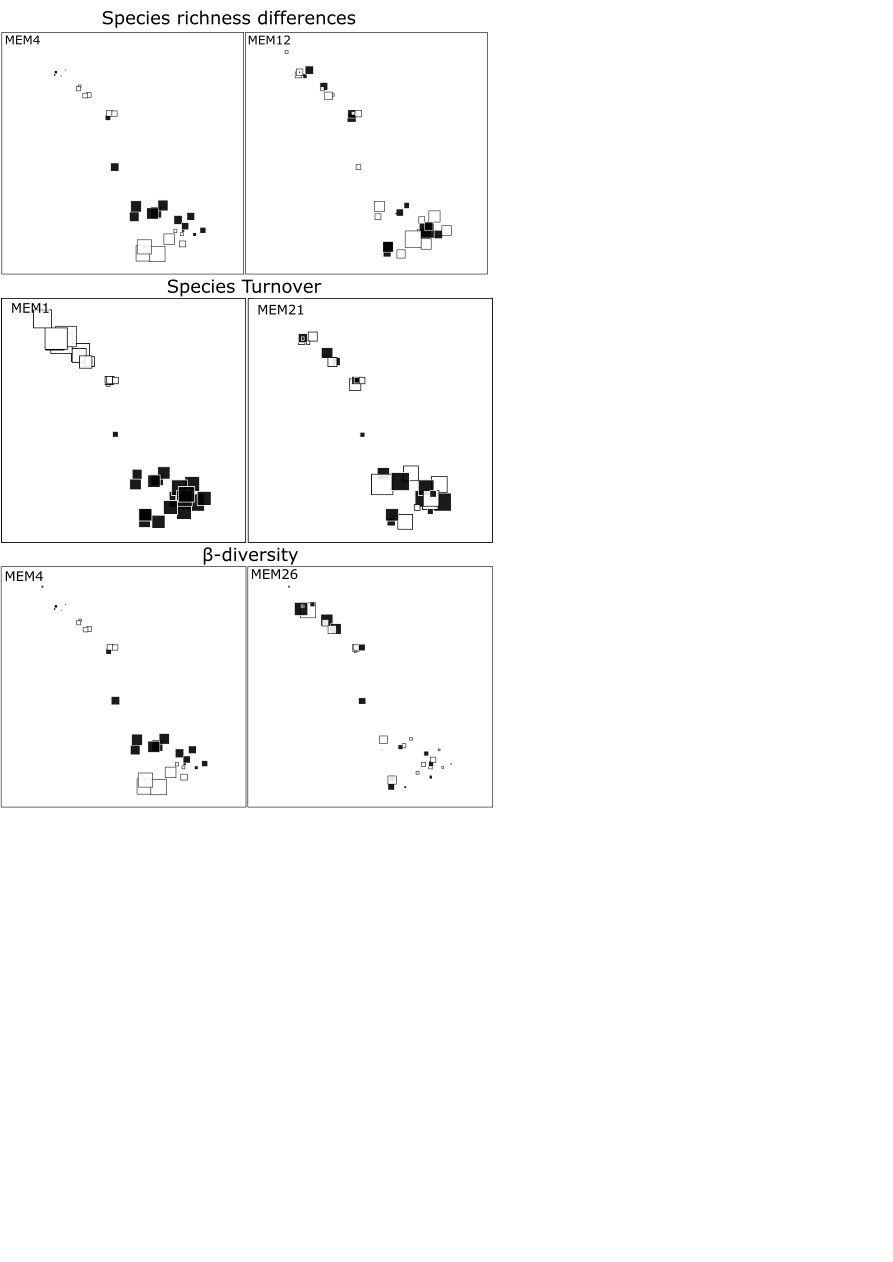
**Supporting S1: Figure S5** Schematic representation of the spatial variables selected by the forward selection procedure for the Palaeotropical forest taxonomic β-diversity and its components.

Medium scaleoad scale scale

Broad scaleoad scale scale

Medium scaleoad scale scale

Broad scaleoad scale scale

Fine scaleoad scale scale

Broad scaleoad scale scale

**
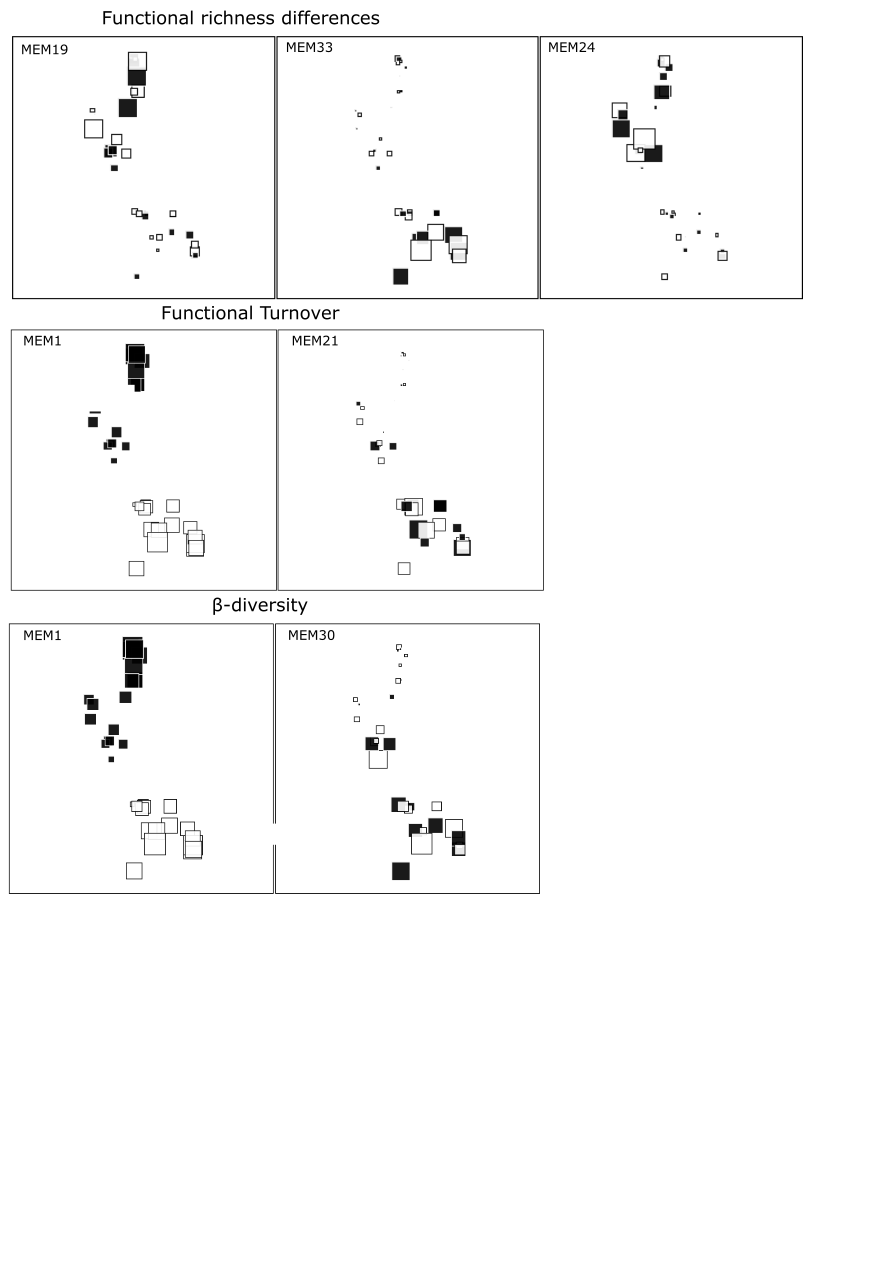
Supporting S1: Figure S6** Schematic representation of the spatial variables selected by the forward selection procedure for the Temperate-Mediterranean forest functional β-diversity and its components.

Fine scaleoad scale scale

Fine scaleoad scale scale

Medium scaleoad scale scale

Fine scaleoad scale scale

Medium scaleoad scale scale

Medium scaleoad scale scale

Broad scaleoad scale scale

Fine scaleoad scale scale

Broad scaleoad scale scale

**Supporting S1: Figure S7** Schematic representation of the spatial variables selected by the forward selection procedure for the Neotropical forest functional β-diversity and its components.
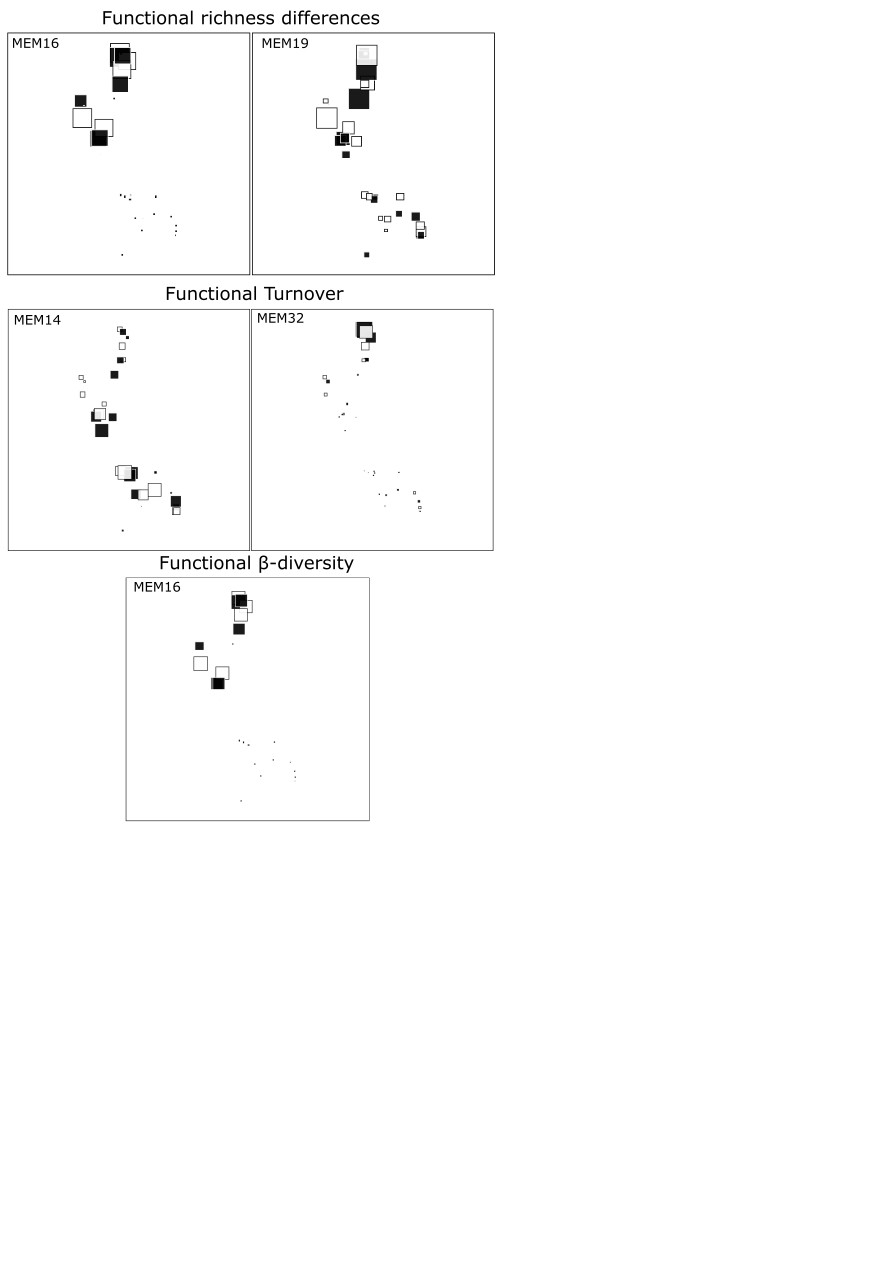


Fine scaleoad scale scale

Medium scaleoad scale scale

Medium scaleoad scale scale

Medium scaleoad scale scale

Medium scaleoad scale scale

**Supporting S1: Figure S8** Schematic representation of the spatial variables selected by the forward selection procedure for the Palaeotropical forest functional β-diversity and its components.


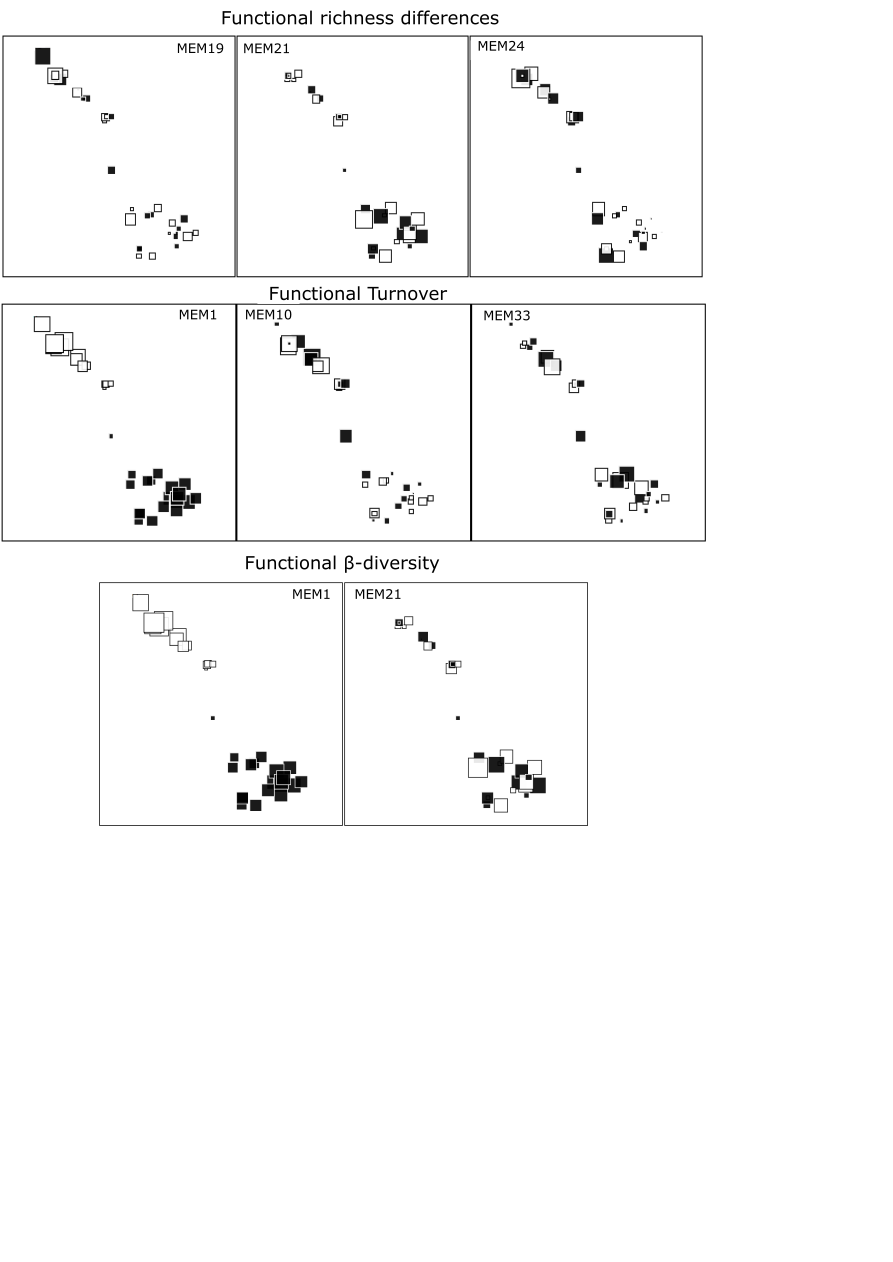


Medium scaleoad scale scale

Broad scaleoad scale scale

Medium scaleoad scale scale

Medium scaleoad scale scale

Medium scaleoad scale scale

Fine scaleoad scale scale

Broad scaleoad scale scale

Broad scaleoad scale scale

**References**

NASA Langley Research Center (LaRC) POWER Project. (2024). Prediction of Worldwide Energy Resource (POWER) Project. Available at: [https://power.larc.nasa.gov/](https://power.larc.nasa.gov/?utm_source=chatgpt.com). Last accessed 07 October 2025.
